# Supplementary material for: Applying Conceptual and Theoretical Frameworks to Health Professions Education Research: An Introductory Workshop
Source: MedEdPORTAL. 2022 Dec 2;18:11286. doi: 10.15766/mep_2374-8265.11286 (PMC9715823; doi:10.15766/mep_2374-8265.11286)
Supplement: Supplementary file 1 — Workshop Slides.pptxFacilitators’ Guide.docxParticipant Worksheet.docxWorkshop Evaluation.docx [file mep_2374-8265.11286-s001.zip › A. Workshop Slides.pptx]

## Slide 1
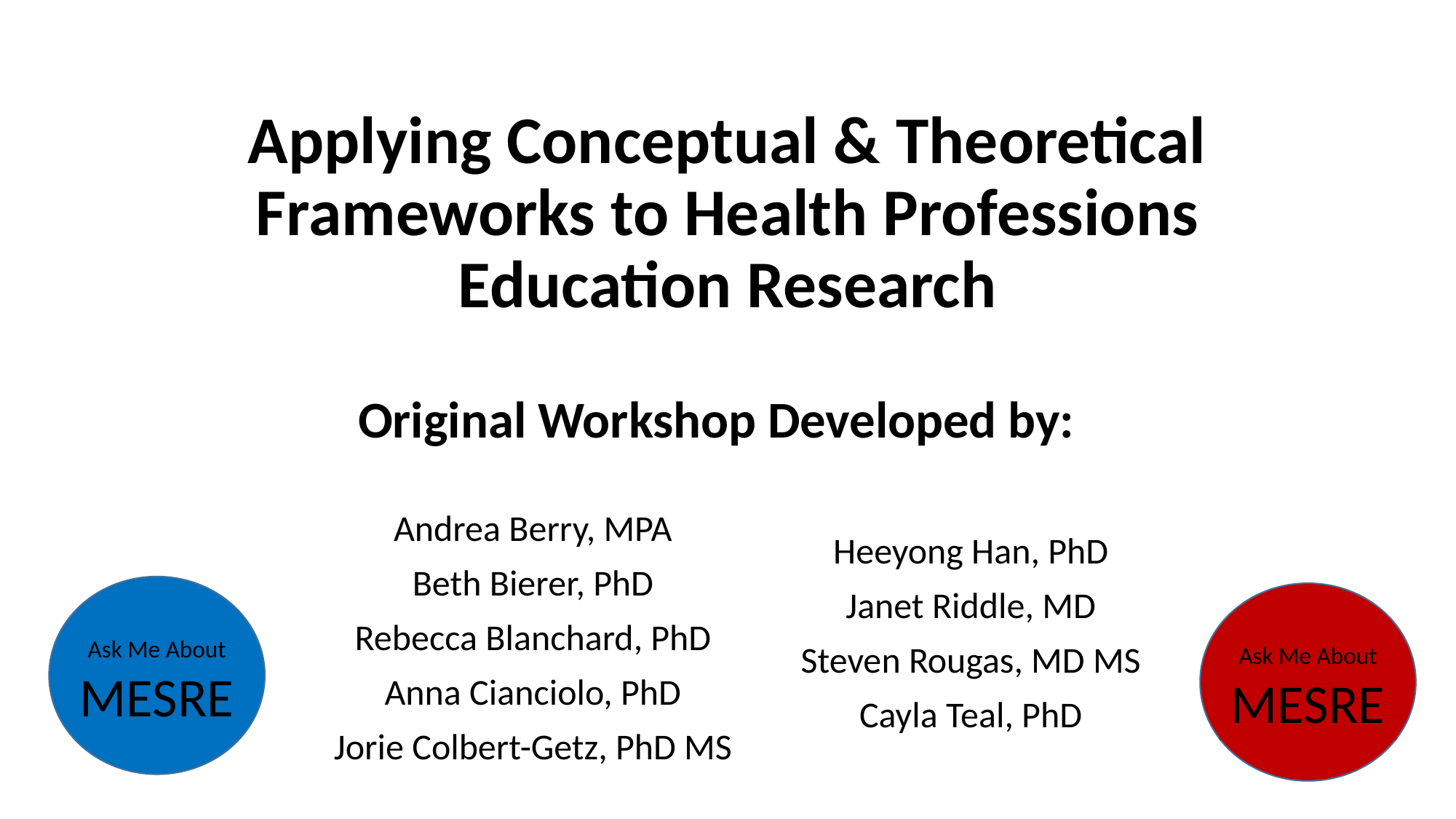

Applying Conceptual & Theoretical Frameworks to Health Professions Education Research
# Original Workshop Developed by:
Heeyong Han, PhD
Janet Riddle, MD
Steven Rougas, MD MS
Cayla Teal, PhD
Andrea Berry, MPA
Beth Bierer, PhD
Rebecca Blanchard, PhD
Anna Cianciolo, PhD
Jorie Colbert-Getz, PhD MS
Ask Me About
MESRE
Ask Me About
MESRE

## Slide 2
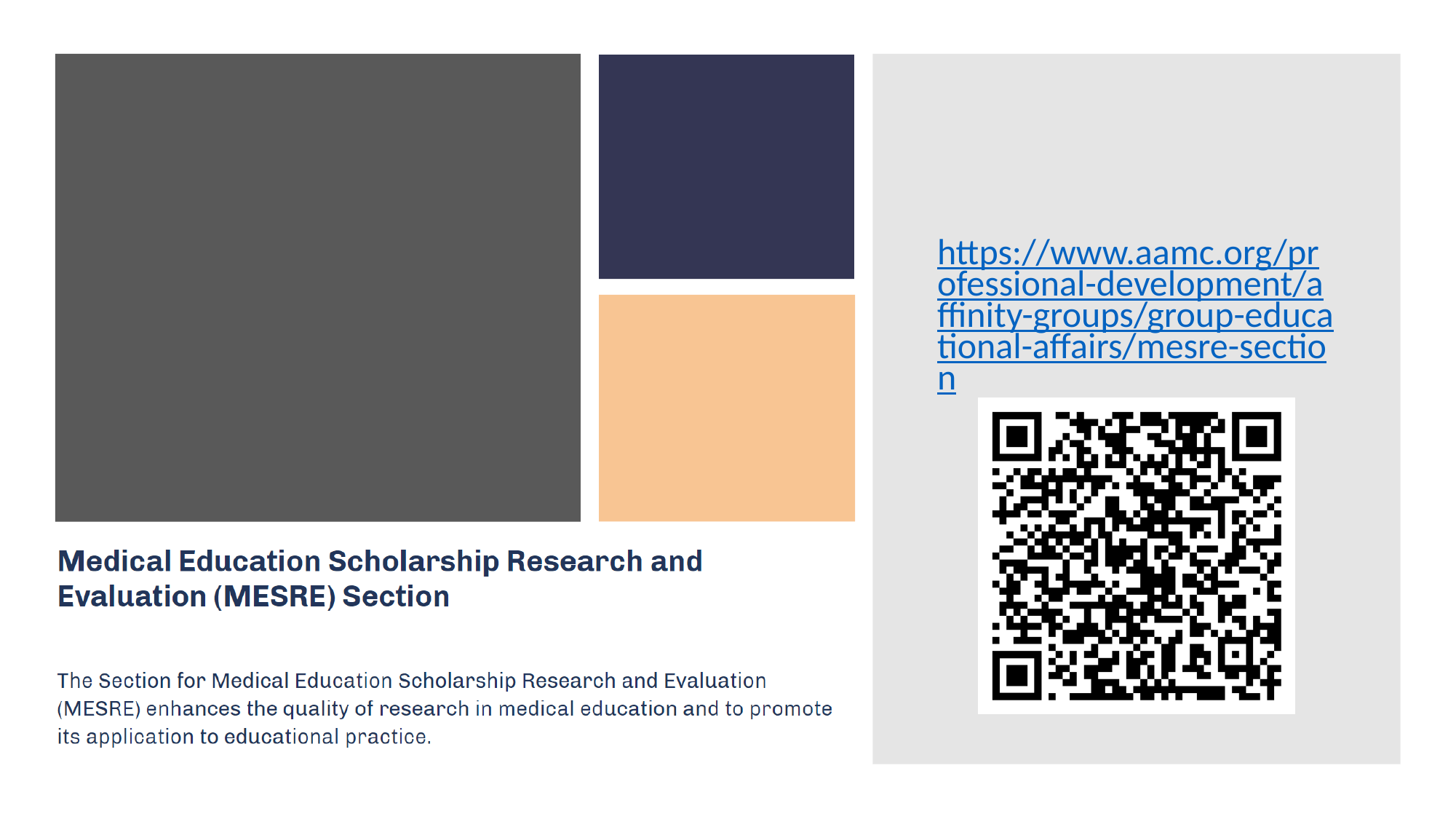

https://www.aamc.org/professional-development/affinity-groups/group-educational-affairs/mesre-section

## Slide 3
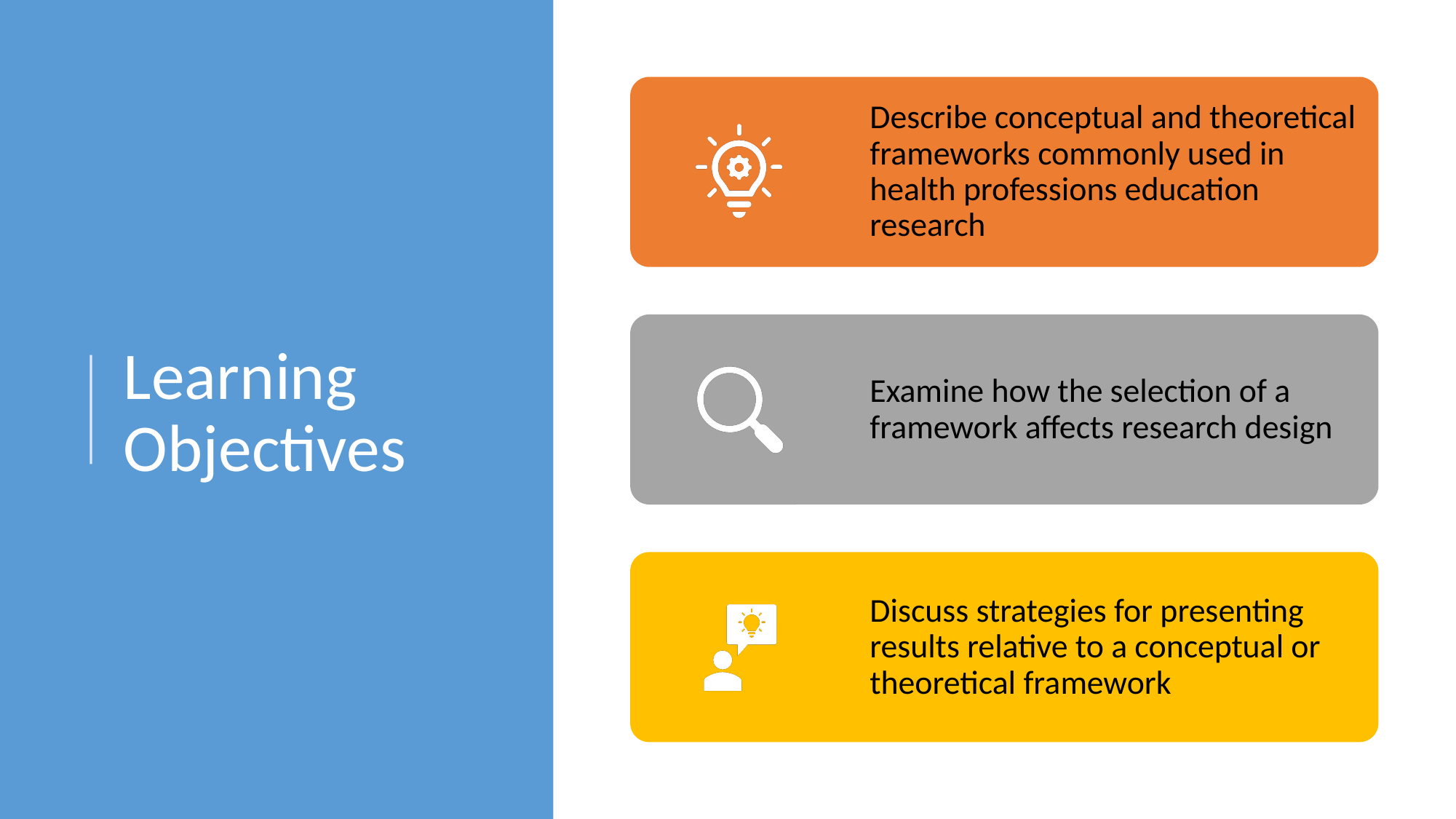

Describe conceptual and theoretical frameworks commonly used in health professions education research
Examine how the selection of a framework affects research design
Discuss strategies for presenting results relative to a conceptual or theoretical framework
# Learning Objectives

## Slide 4
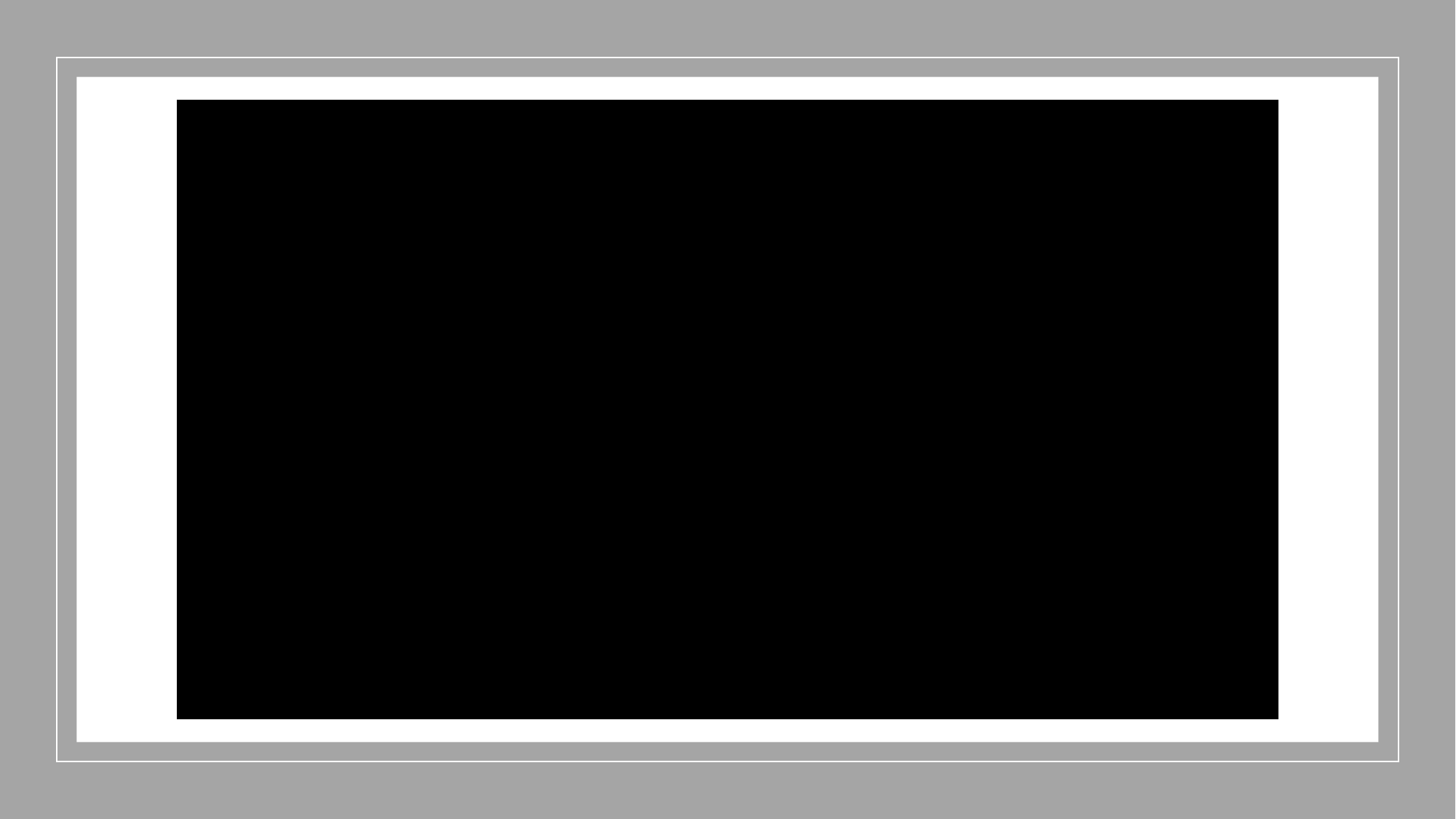

## Slide 5
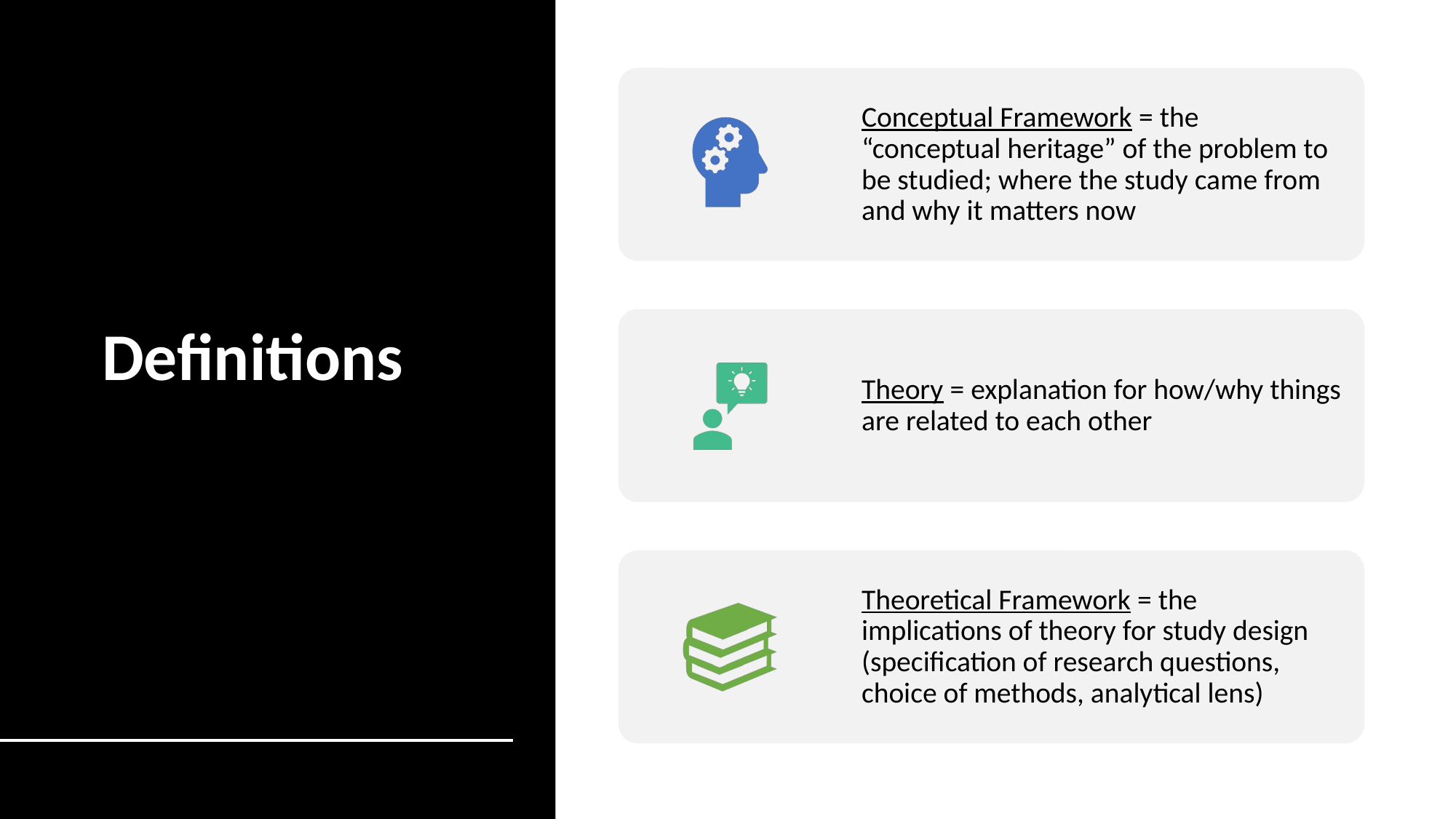

# Definitions
Conceptual Framework = the “conceptual heritage” of the problem to be studied; where the study came from and why it matters now
Theory = explanation for how/why things are related to each other
Theoretical Framework = the implications of theory for study design (specification of research questions, choice of methods, analytical lens)

## Slide 6
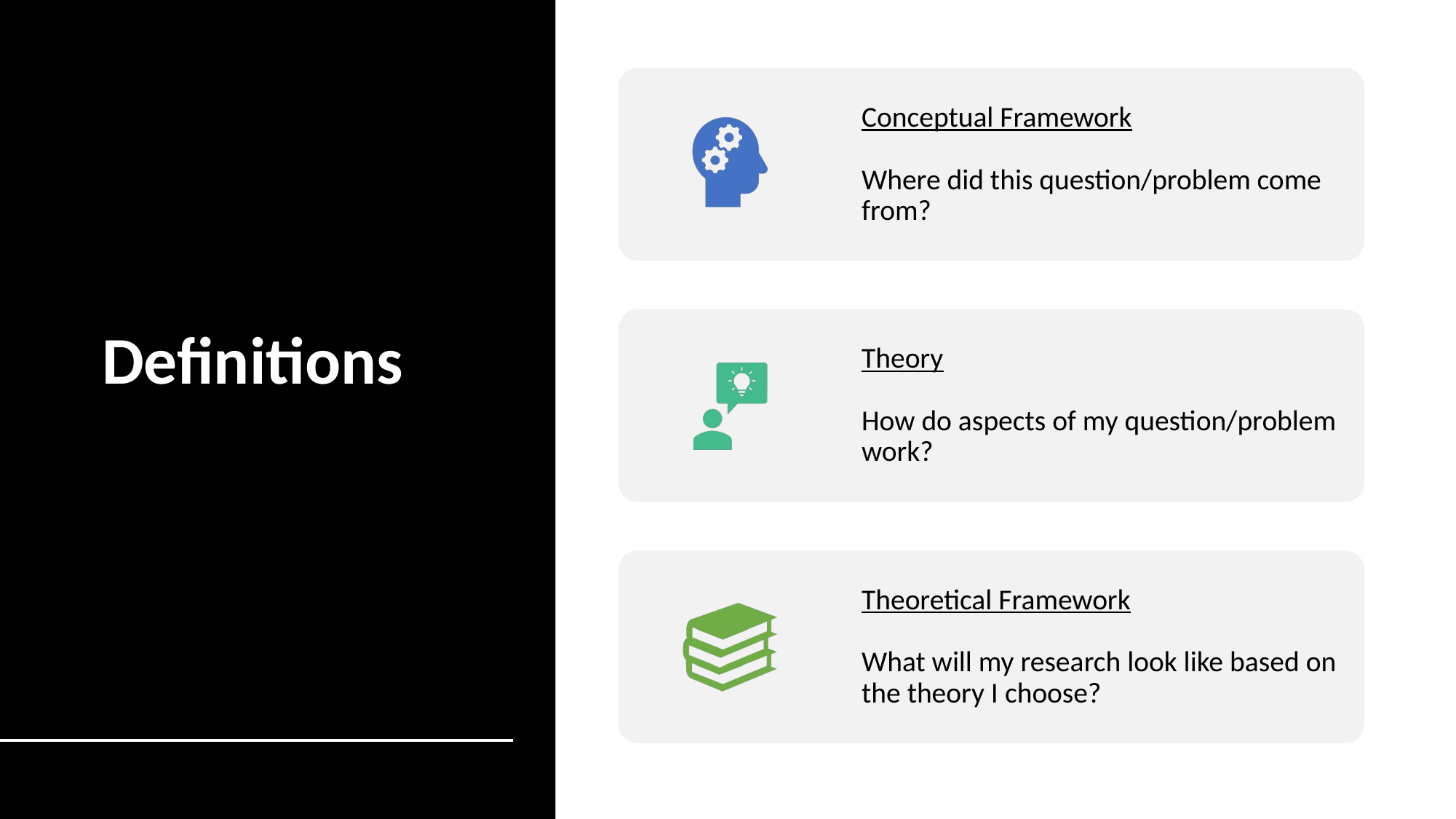

# Definitions
Conceptual Framework
Where did this question/problem come from?
Theory
How do aspects of my question/problem work?
Theoretical Framework
What will my research look like based on the theory I choose?

## Slide 7
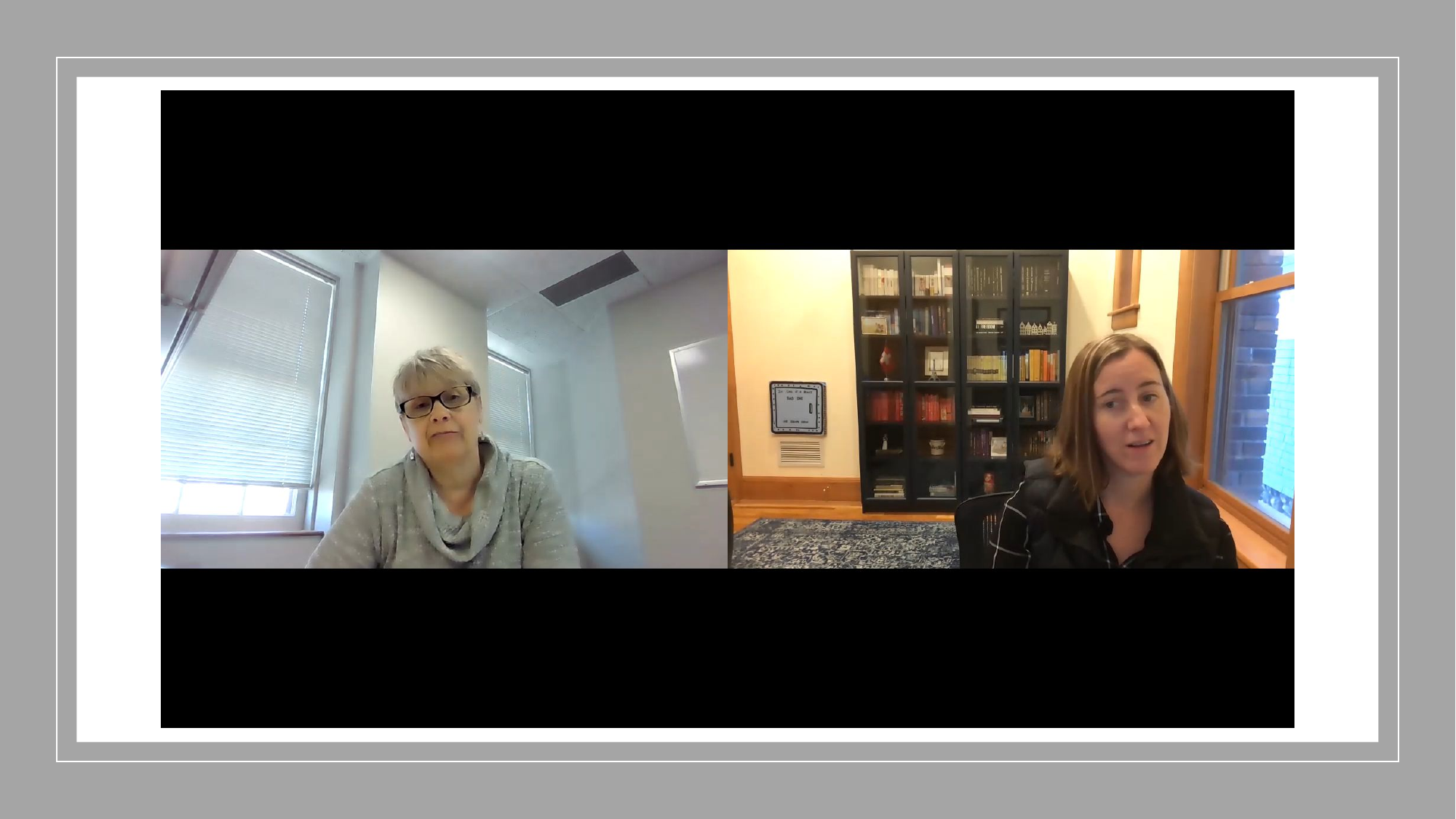

## Slide 8
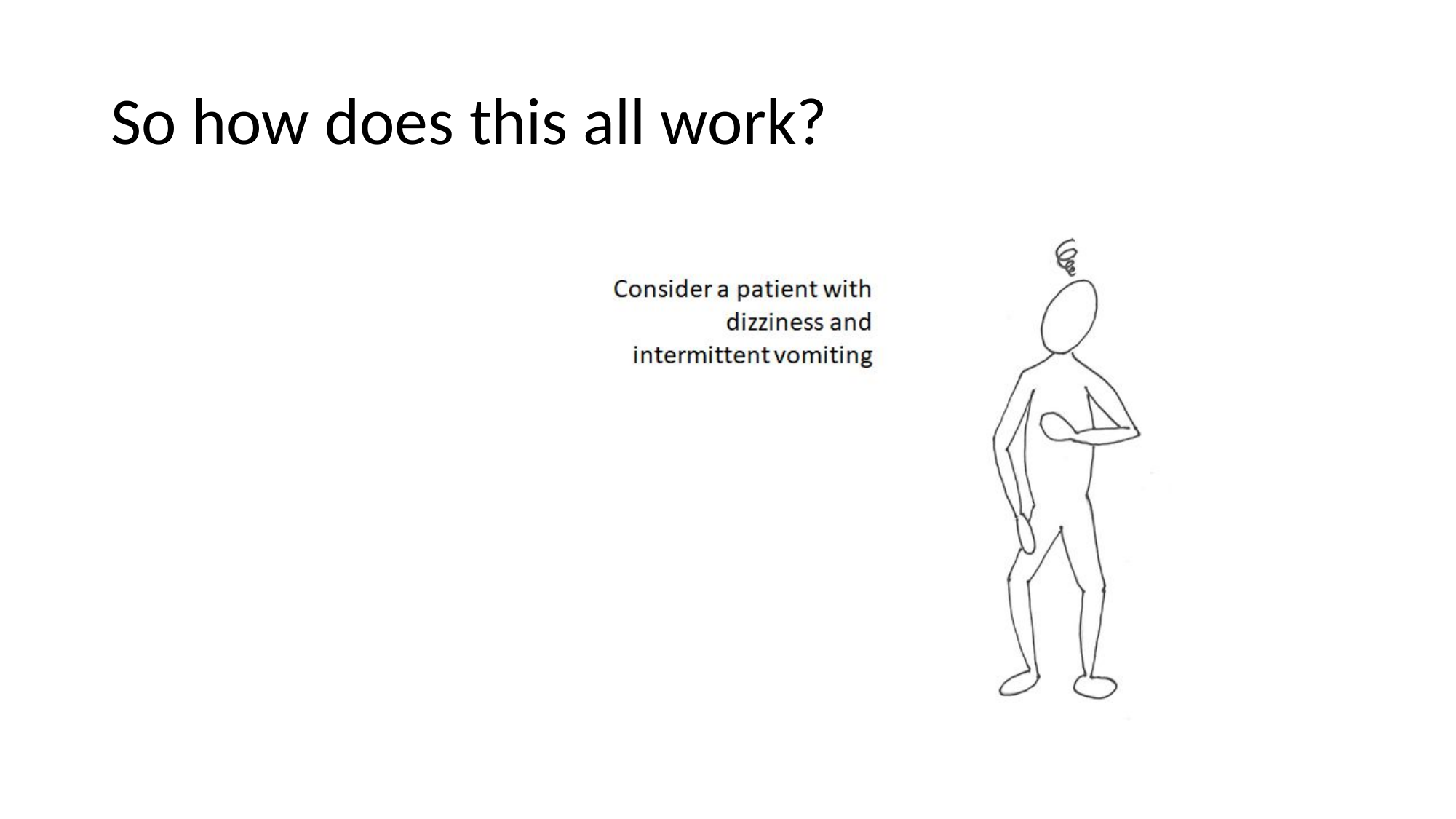

# So how does this all work?

## Slide 9
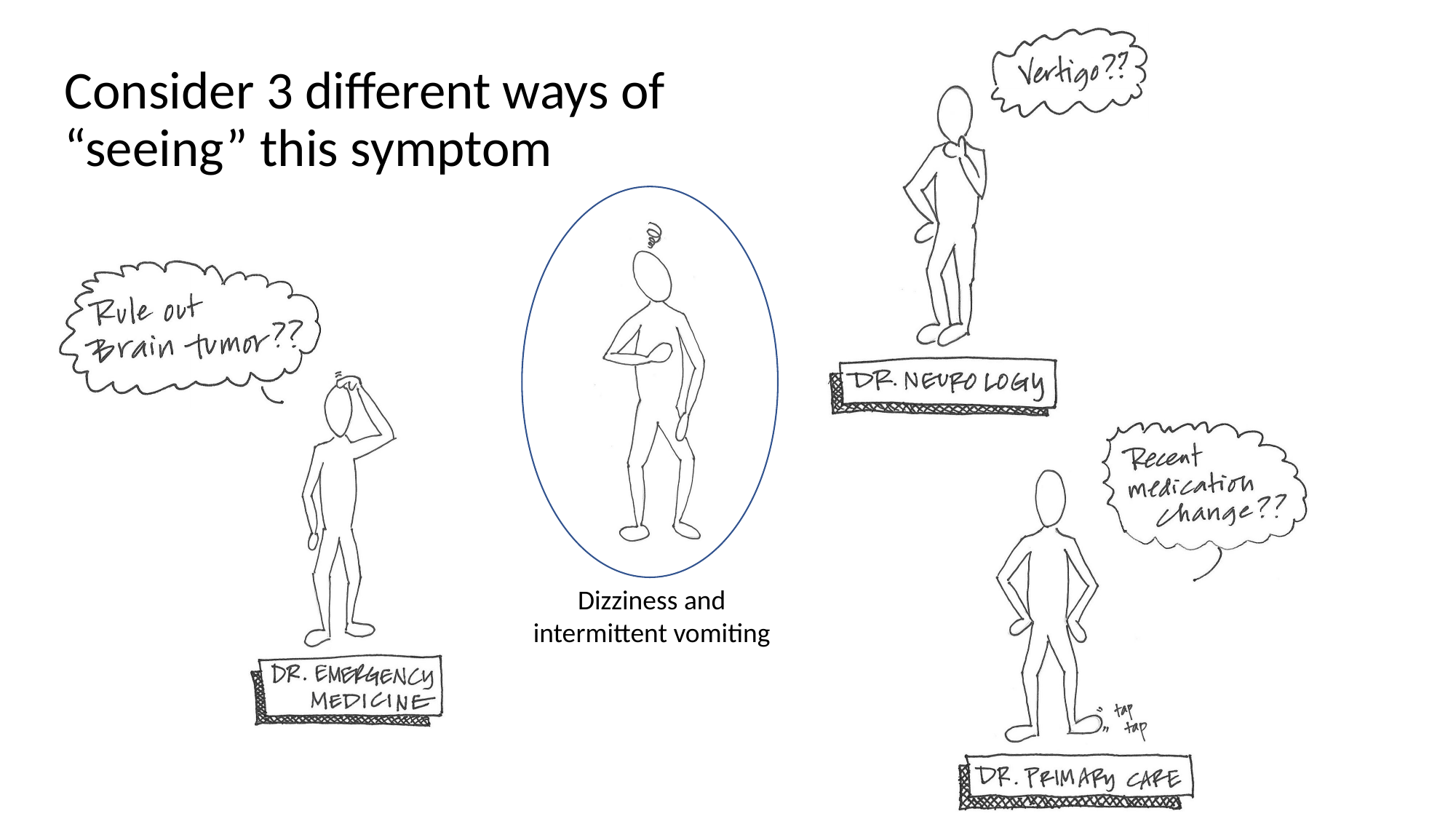

# Consider 3 different ways of “seeing” this symptom
Dizziness and intermittent vomiting

## Slide 10
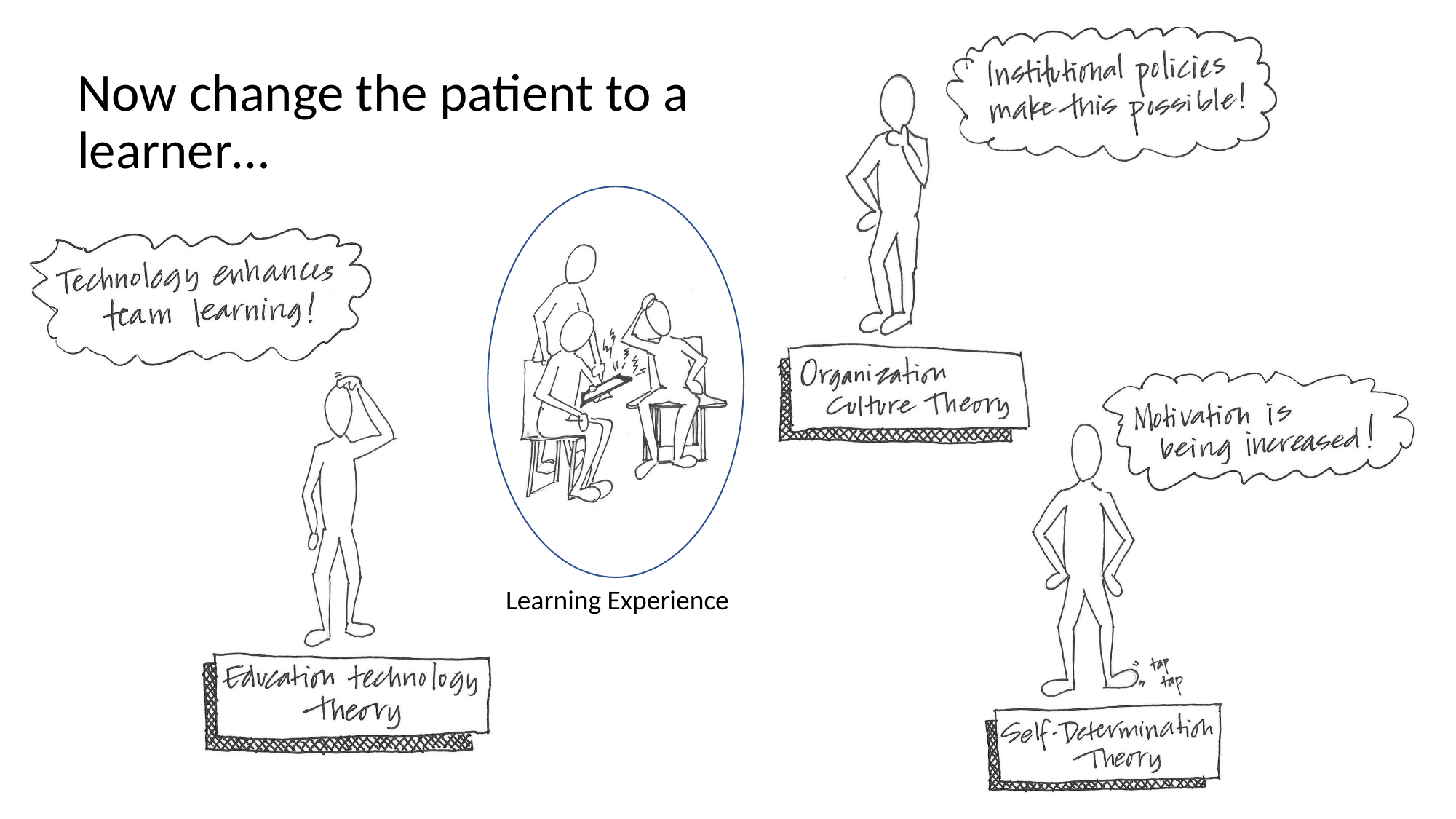

# Now change the patient to a learner…
Learning Experience

## Slide 11
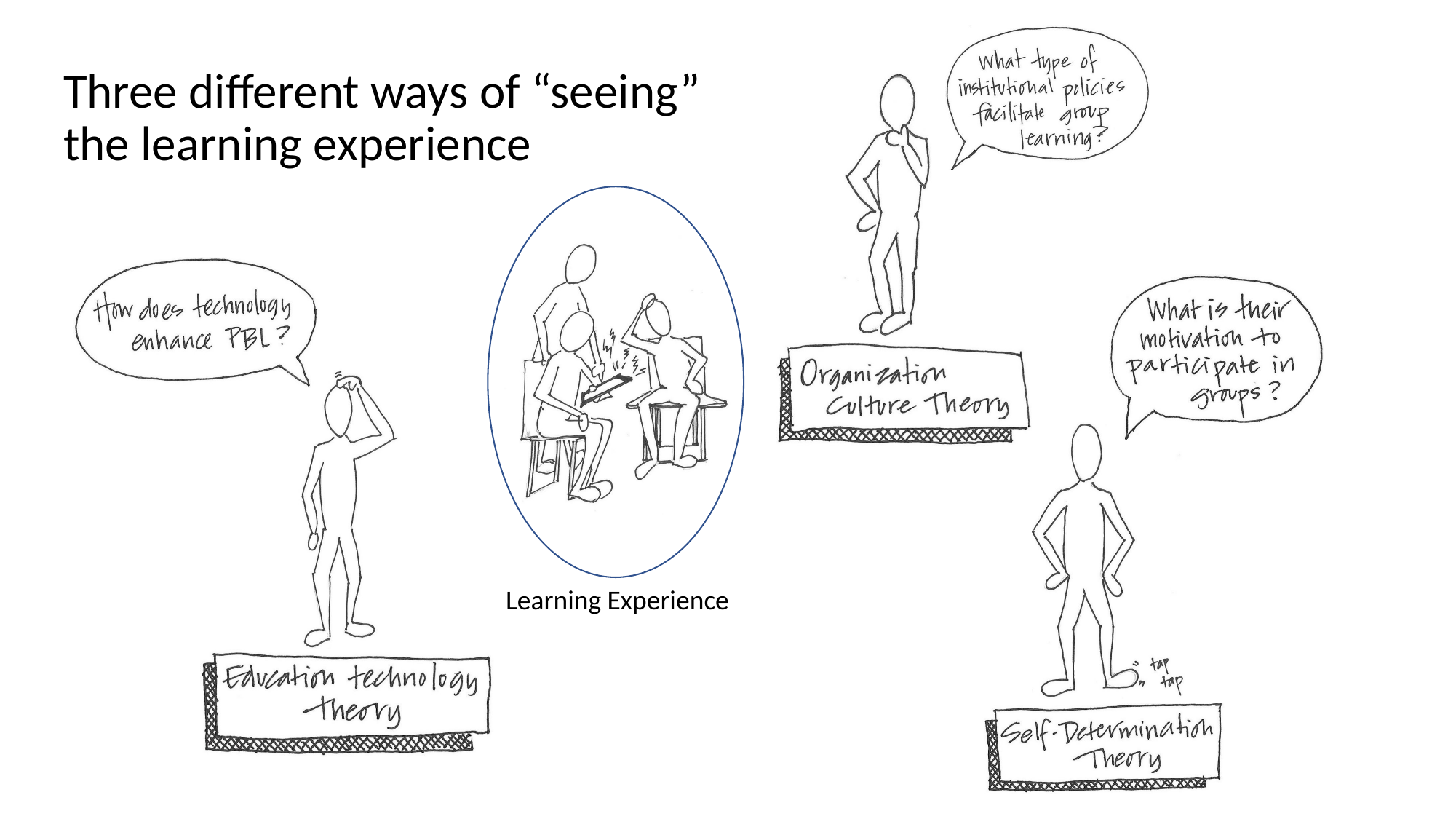

Three different ways of “seeing” the learning experience
Learning Experience

## Slide 12
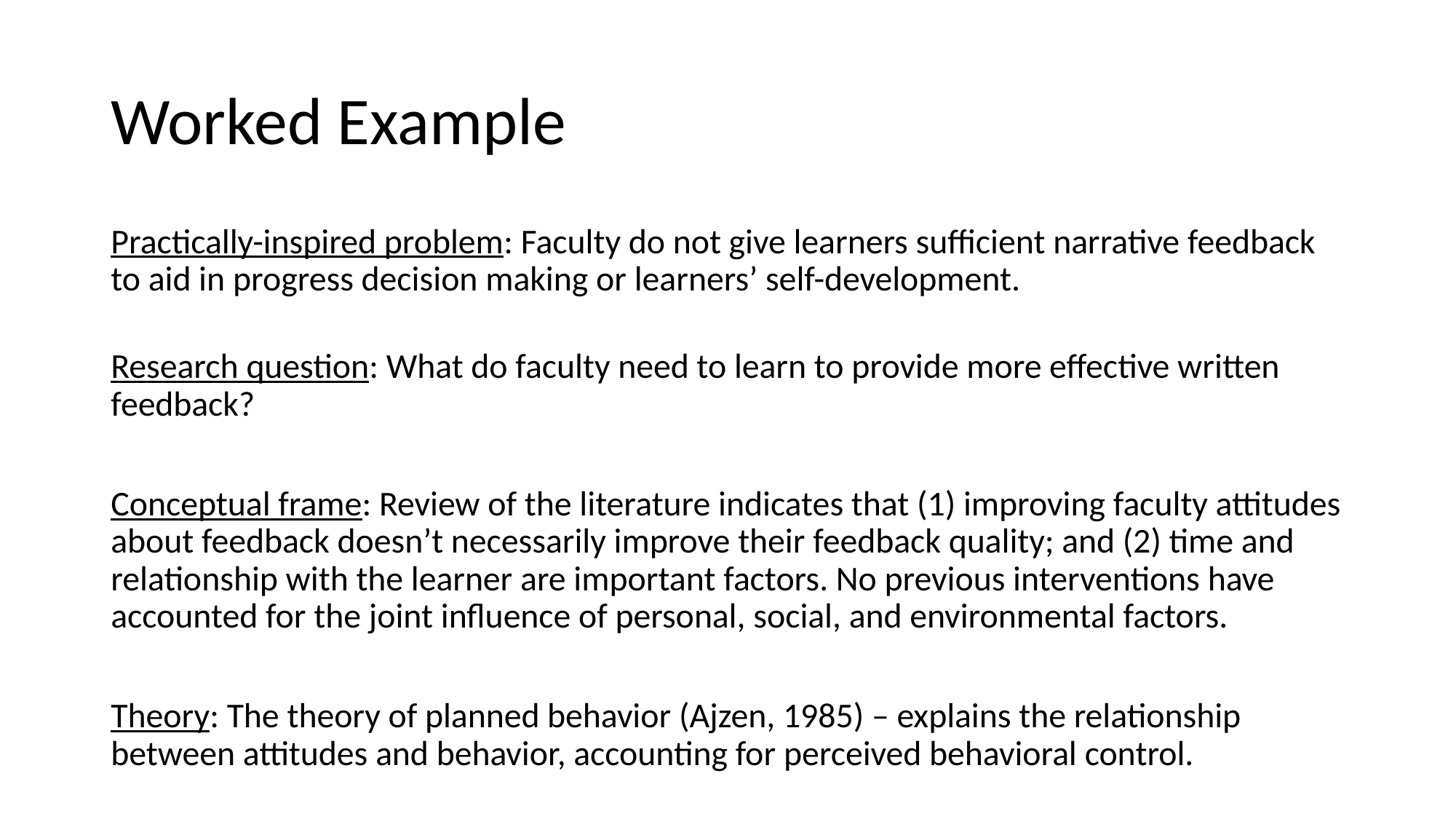

# Worked Example
Practically-inspired problem: Faculty do not give learners sufficient narrative feedback to aid in progress decision making or learners’ self-development.
Research question: What do faculty need to learn to provide more effective written feedback?
Conceptual frame: Review of the literature indicates that (1) improving faculty attitudes about feedback doesn’t necessarily improve their feedback quality; and (2) time and relationship with the learner are important factors. No previous interventions have accounted for the joint influence of personal, social, and environmental factors.
Theory: The theory of planned behavior (Ajzen, 1985) – explains the relationship between attitudes and behavior, accounting for perceived behavioral control.

## Slide 13
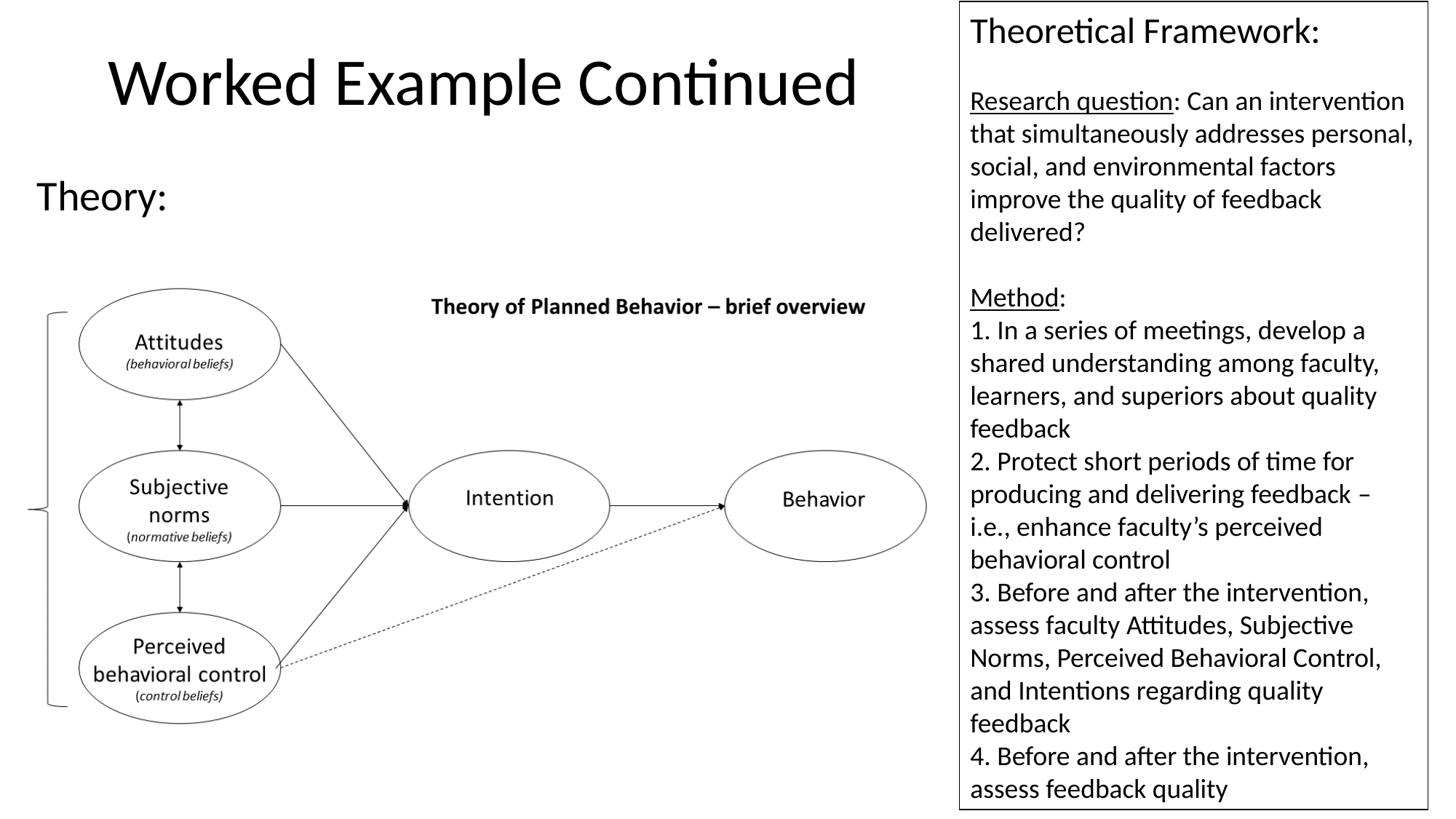

Theoretical Framework:
Research question: Can an intervention that simultaneously addresses personal, social, and environmental factors improve the quality of feedback delivered?
Method:
1. In a series of meetings, develop a shared understanding among faculty, learners, and superiors about quality feedback
2. Protect short periods of time for producing and delivering feedback – i.e., enhance faculty’s perceived behavioral control
3. Before and after the intervention, assess faculty Attitudes, Subjective Norms, Perceived Behavioral Control, and Intentions regarding quality feedback
4. Before and after the intervention, assess feedback quality
# Worked Example Continued
Theory:

## Slide 14
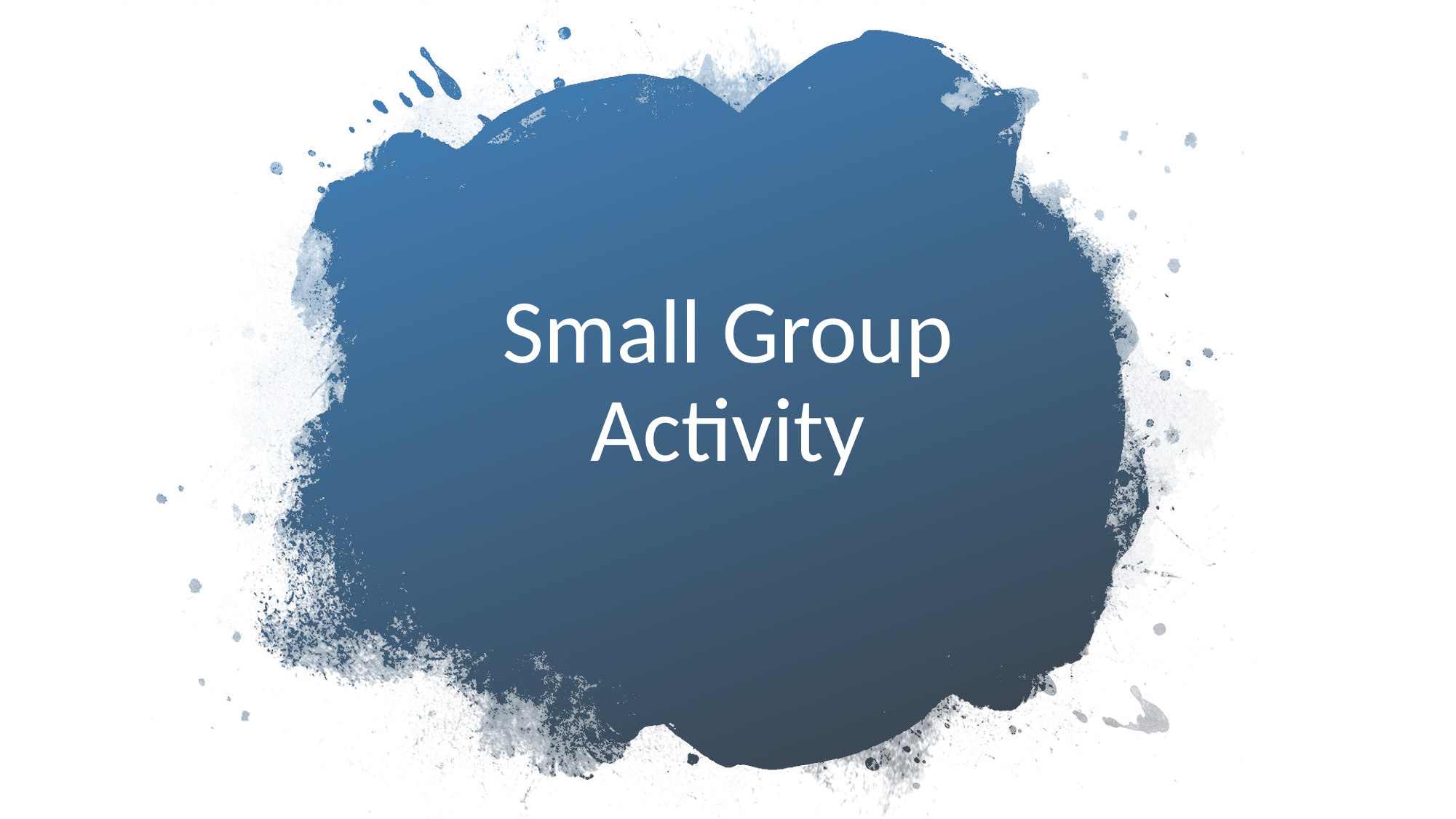

# Small Group Activity

## Slide 15
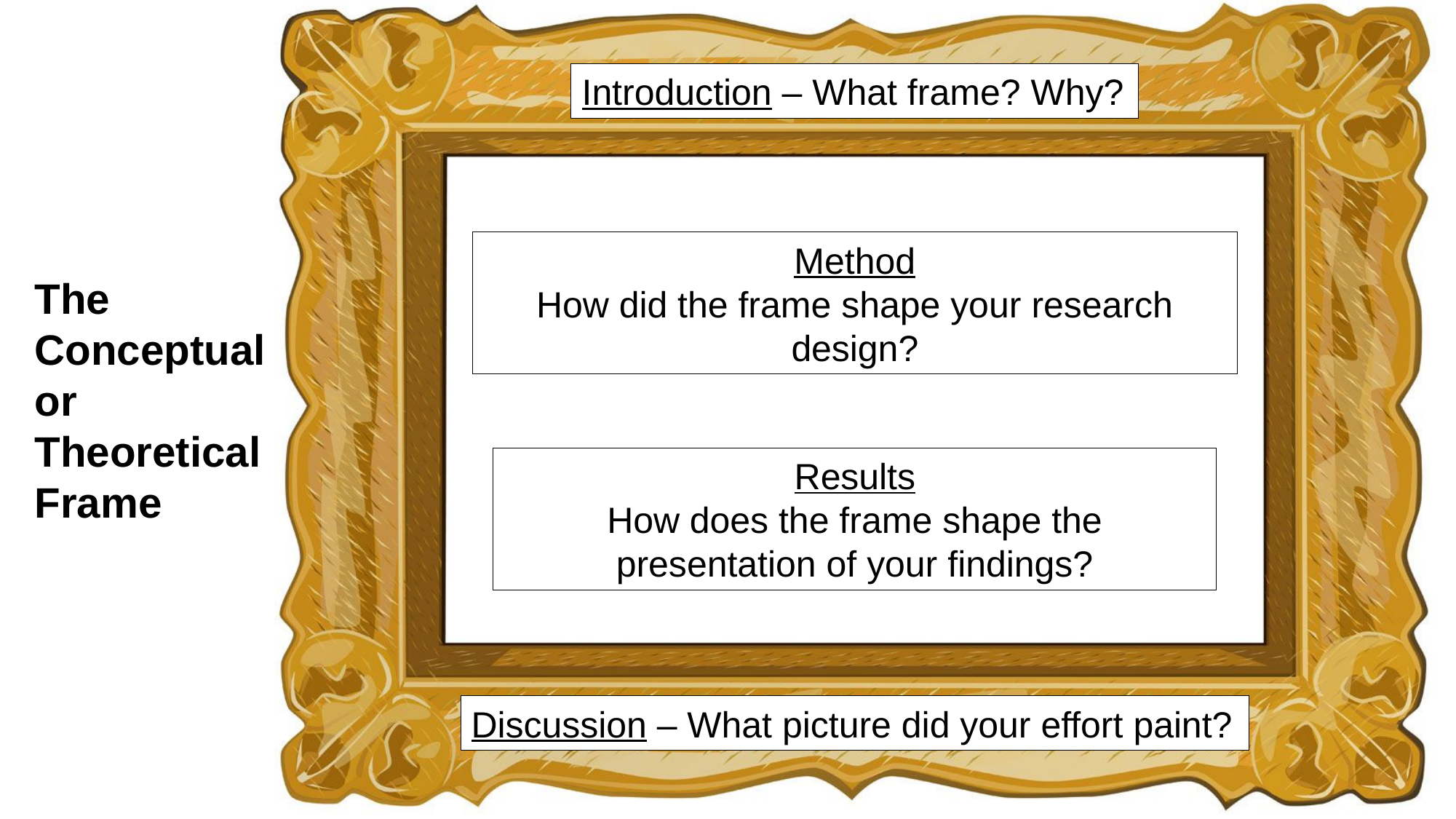

Introduction – What frame? Why?
Method
How did the frame shape your research design?
Results
How does the frame shape the presentation of your findings?
The Conceptual or Theoretical Frame
Discussion – What picture did your effort paint?

## Slide 16
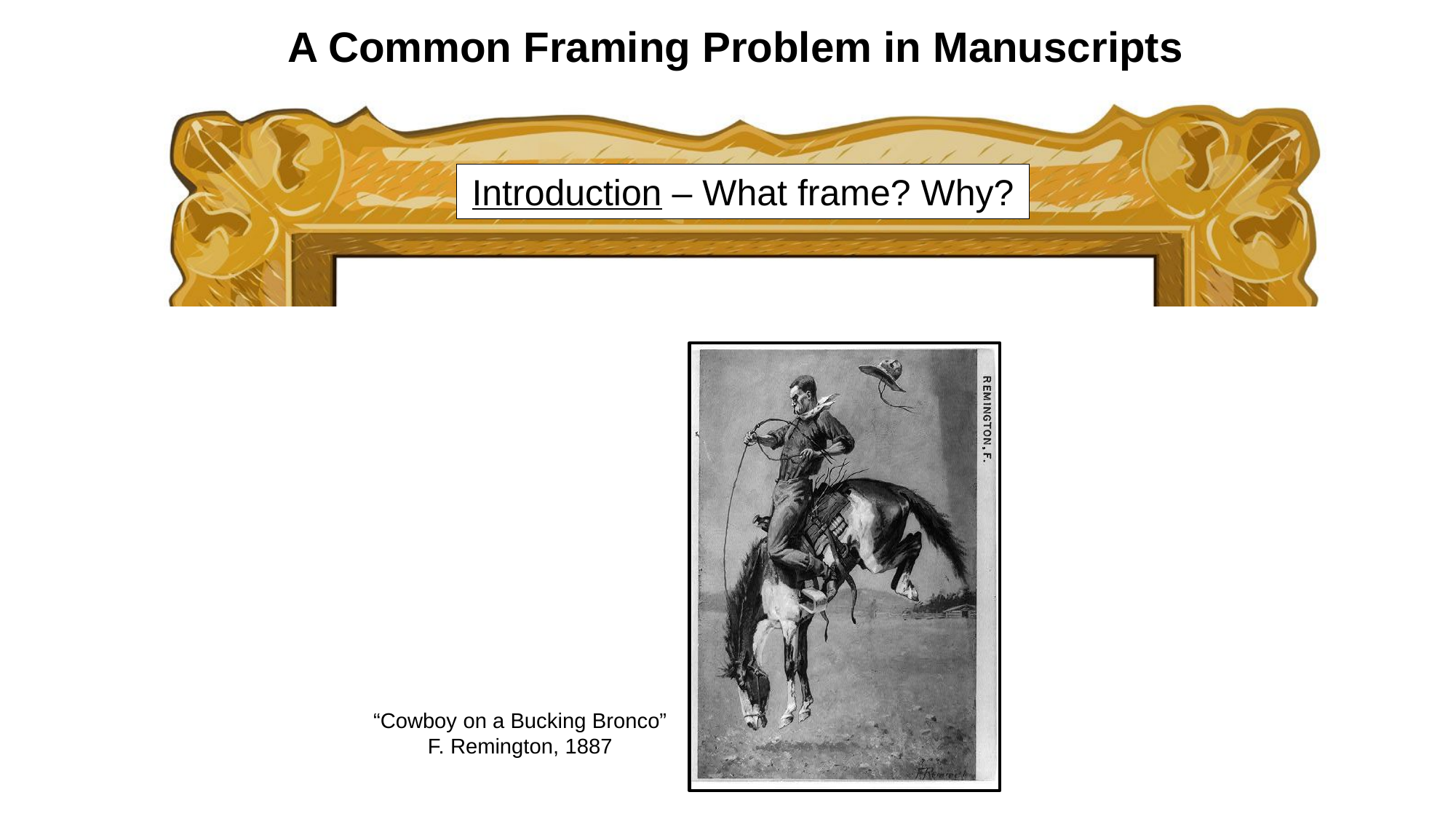

A Common Framing Problem in Manuscripts
Introduction – What frame? Why?
“Cowboy on a Bucking Bronco”
F. Remington, 1887

## Slide 17
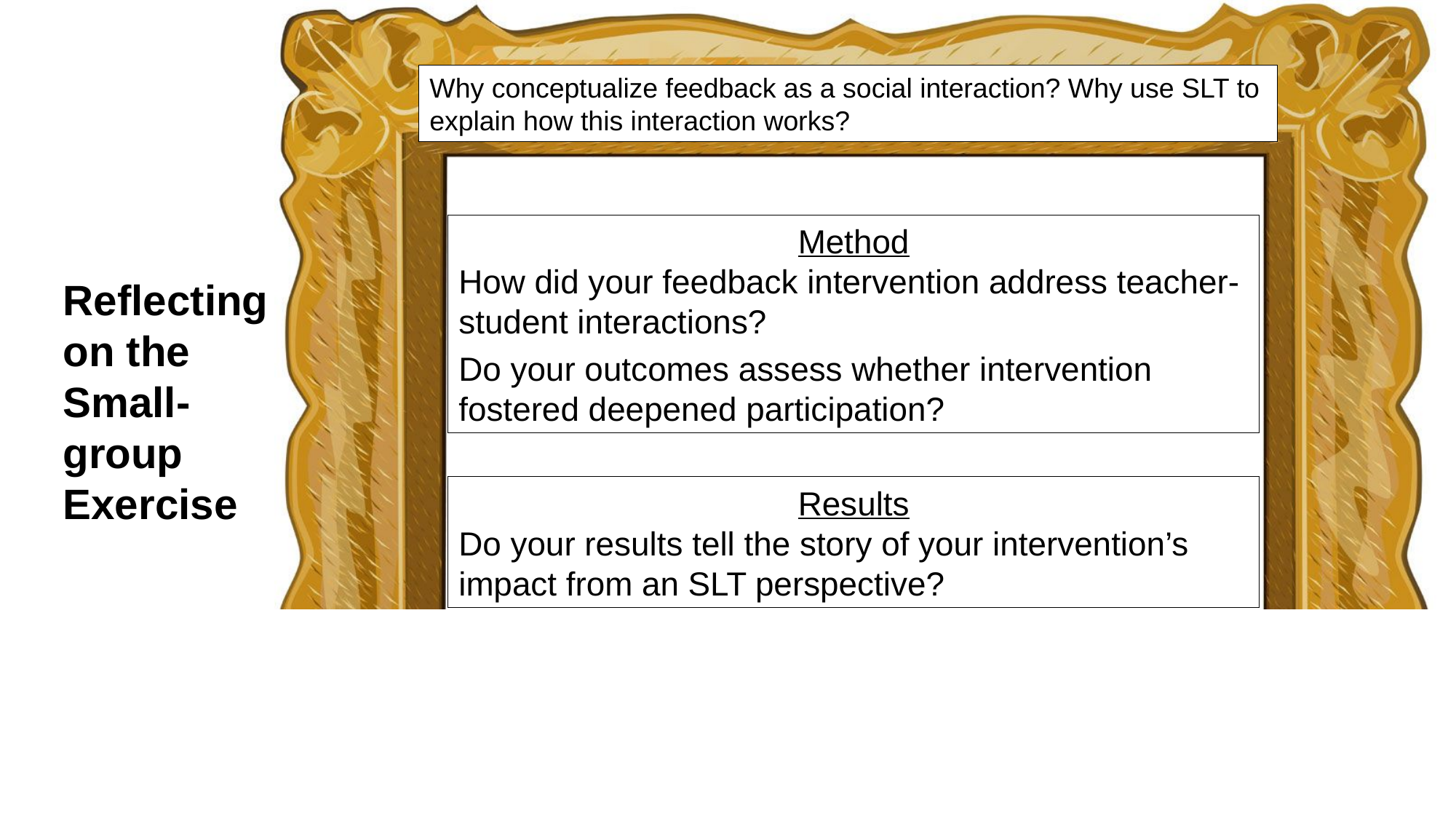

Why conceptualize feedback as a social interaction? Why use SLT to explain how this interaction works?
Method
How did your feedback intervention address teacher-student interactions?
Do your outcomes assess whether intervention fostered deepened participation?
Reflecting
on the
Small-group
Exercise
Results
Do your results tell the story of your intervention’s impact from an SLT perspective?

## Slide 18
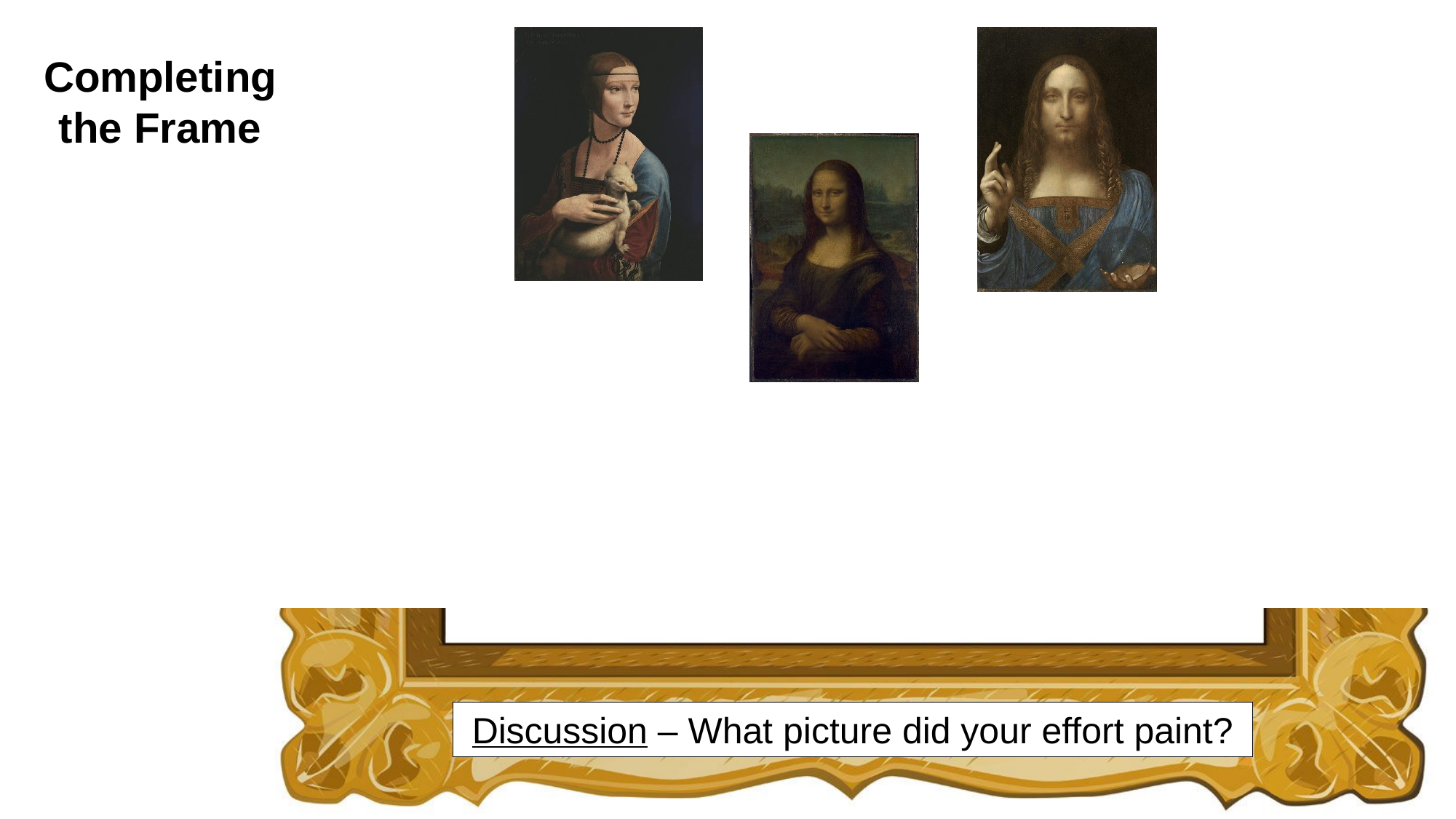

Completing the Frame
Discussion – What picture did your effort paint?

## Slide 19
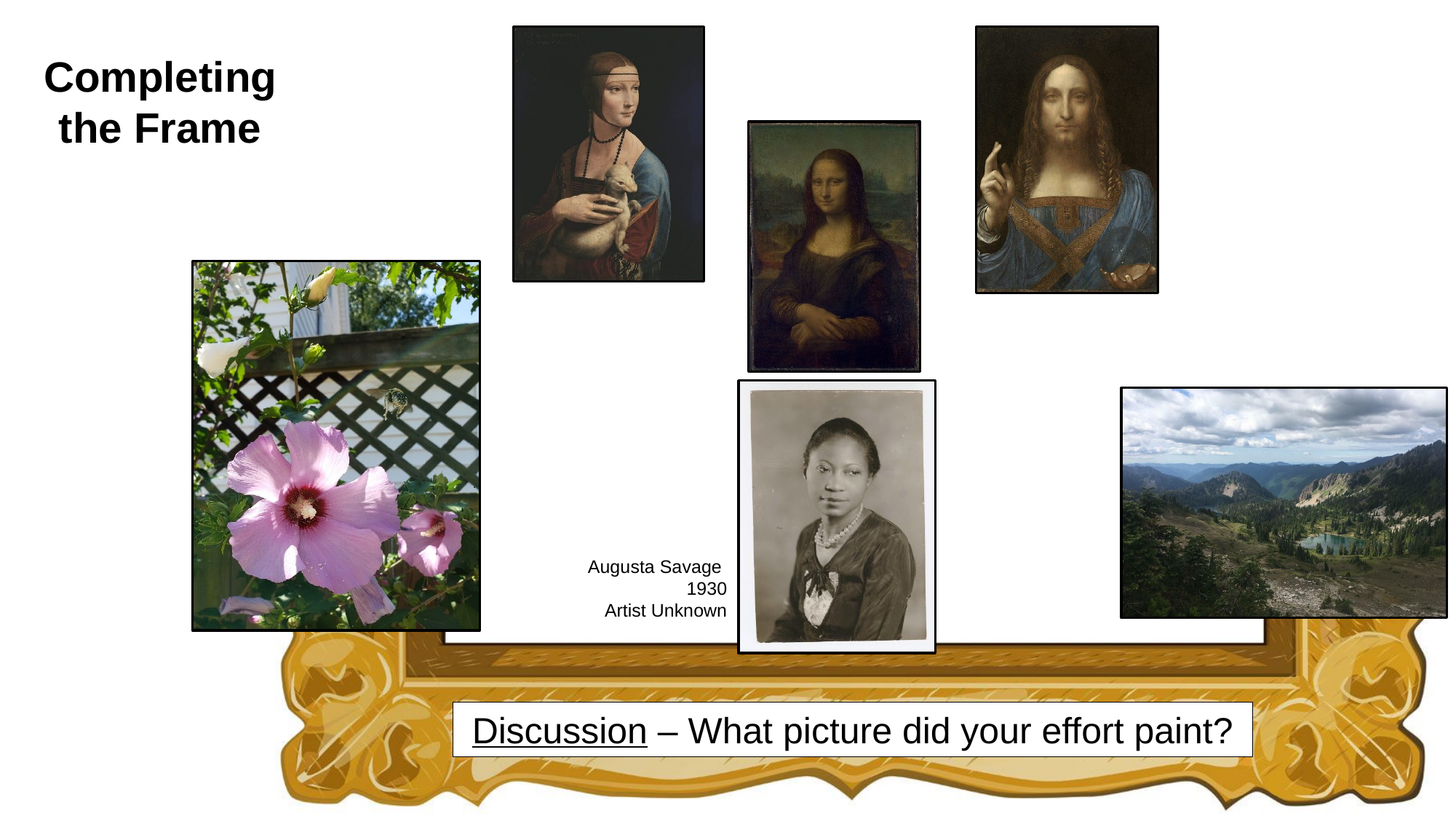

Completing the Frame
Augusta Savage
1930
Artist Unknown
Discussion – What picture did your effort paint?

## Slide 20
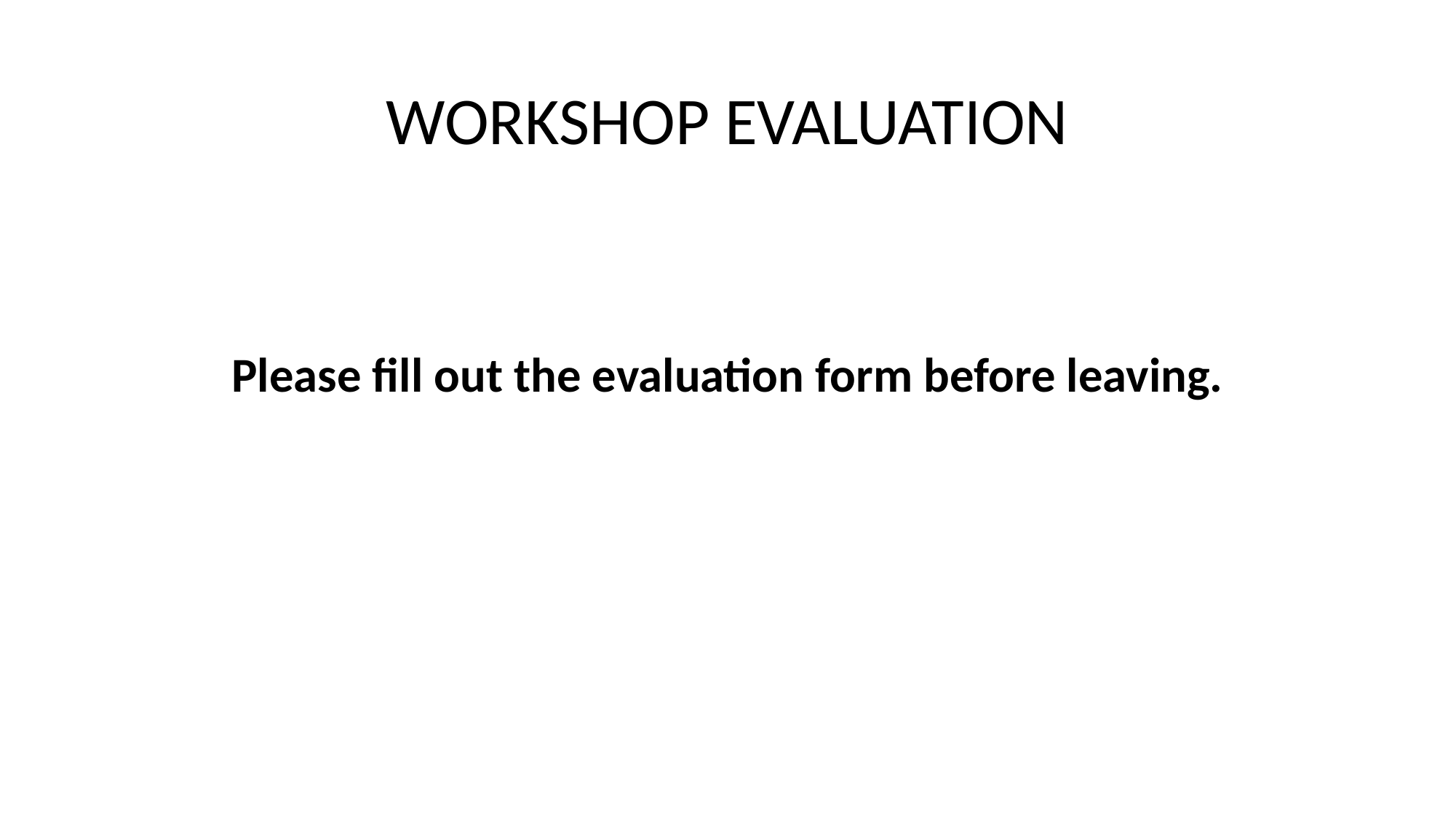

# WORKSHOP EVALUATION
Please fill out the evaluation form before leaving.

## Slide 21
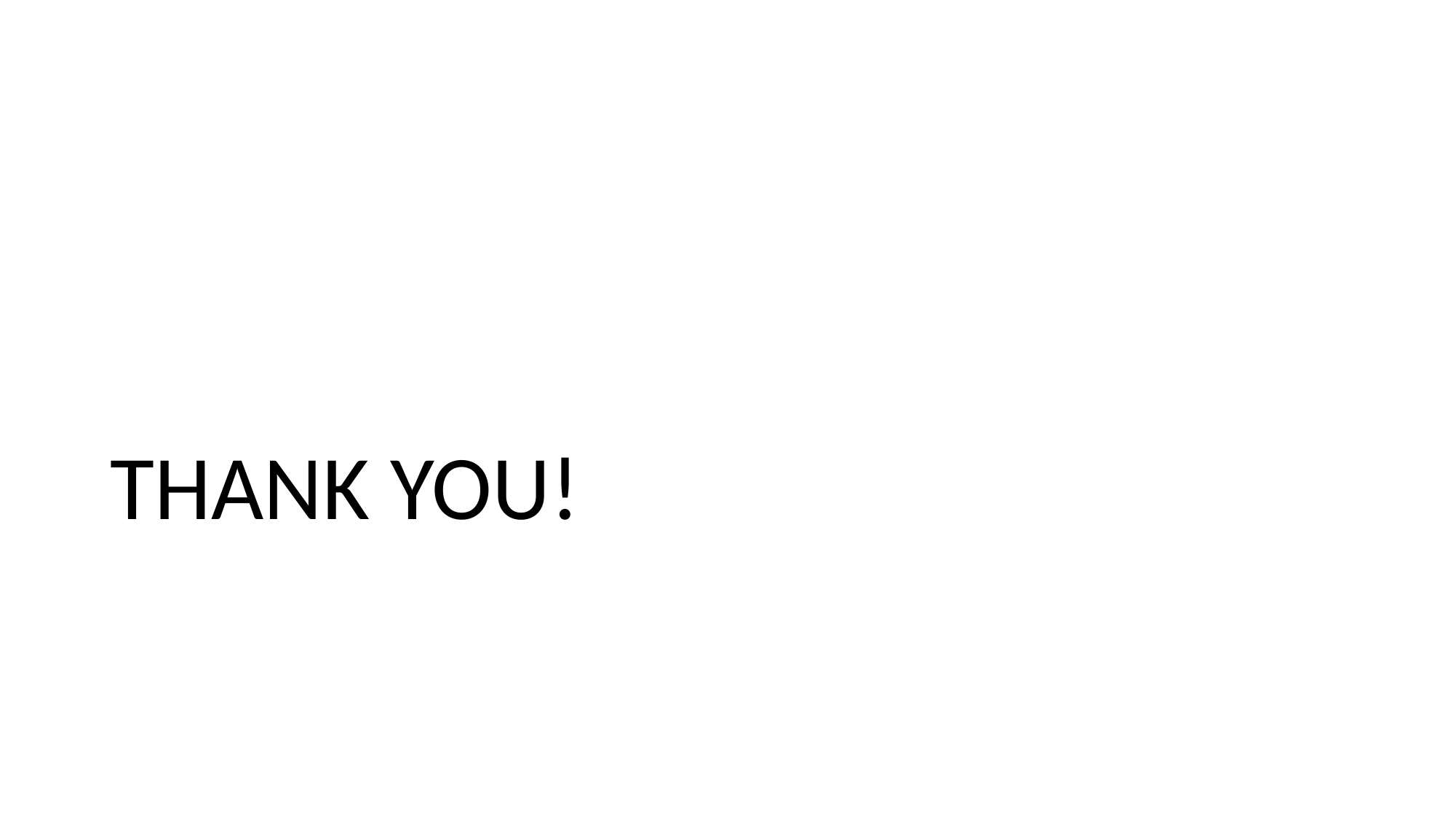

# THANK YOU!
