# Supplementary material for: Applying Conceptual and Theoretical Frameworks to Health Professions Education Research: An Introductory Workshop
Source: MedEdPORTAL. 2022 Dec 2;18:11286. doi: 10.15766/mep_2374-8265.11286 (PMC9715823; doi:10.15766/mep_2374-8265.11286)
Supplement: Supplementary file 1 — Workshop Slides.pptxFacilitators’ Guide.docxParticipant Worksheet.docxWorkshop Evaluation.docx [file mep_2374-8265.11286-s001.zip › B. Facilitators Guide.docx]

**Facilitators’ Guide**

*This workshop may be delivered in-person or virtually and includes both large group and small group (breakout) activities. It is recommended to have one lead facilitator for the entire session, plus one small group facilitator for each group of 5-10 participants.*

*Facilitators should review the PowerPoint slides and speaker notes in advance of the session.*

**Workshop Overview**

*PowerPoint slides 1-14 are presented to all participants for the first 15-20 minutes of the session. The small group activity then takes approximately 25-30 minutes and, finally, the large group debriefing (including narrated PowerPoint slides 15-19) concludes the session. Times may be adjusted based on the size of the group. At the workshop's end, facilitators should remind participants to complete the session evaluation.*

**Small Group Activity: Facilitator Instructions**

*We recommend that participants form small groups of 5-10 (or, if conducting this workshop virtually, place 5-10 participants in breakout rooms) for this 30-minute small group activity.*

*The purpose of the small group activity is to help participants feel more comfortable applying a theory to a common health professions education (HPE) problem. Participants will likely still have questions when they leave this small group activity: however, they will have seen the application of a theory to a (HPE) problem twice during this session - once during the PowerPoint presentation and again during the small group activity. The goal is that participants begin to recognize questions that they may ask a research mentor and/or that help them become more educated consumers of HPE research literature.*

*Small group facilitators begin the session by reviewing the key features of Situated Learning Theory (SLT) summarized on page 2.*

*Then, facilitators use the Small Group Activity Template to guide participants in refining the research question provided using SLT and help participants consider how SLT can guide research design including participants, setting, data sources, and data collection strategies.*

*Before the small group activity concludes, each small group facilitator asks for any remaining questions or tips the participants want to share with the larger group and records these responses for subsequent large group discussion. In addition, if participants want further exploration into SLT as a theoretical frame, they might work with a librarian to identify articles where it has been used as a framework in published HPE research (page 2 includes one example).*

**Situated Learning Theory**

**Brief description**

Situated Learning Theory (SLT) was developed by Jean Lave and Etienne Wenger (1991), who offered a perspective on how people learn that differs from traditional individualistic and cognitivist ways of conceptualizing learning. According to SLT, learning is situated in a local and social context, rather than occurring solely inside a person’s head. Specifically, learning happens within a community of practice in which senior members at the community’s core guide and facilitate the participation of new members on the periphery, and community member development occurs through shared practice. Deepened participation in the community of practice--i.e., learning--is enabled by dialogue and interaction with others and often is unintentional, rather than deliberate.

**Example**

Egan, T., & Jaye, C. (2009). Communities of clinical practice: The social organization of clinical learning. *health: An Interdisciplinary Journal for the Social Study of Health, Illness, and Medicine, 31*(1): 107-125. DOI:10.1177/1363459308097363.

*“The social organization of clinical learning is under-theorized in the sociological literature on the social organization of health care. Professional scopes of practice and jurisdictions are formally defined by professional principles and standards and reflected in legislation; however, these are mediated through the day-to-day clinical activities of social groupings of clinical teams. The activities of health service providers typically occur within communities of clinical practice. These are also major sites for clinical curriculum delivery, where clinical students learn not only clinical skills but also how to be health professionals. In this article, we apply Wenger’s model of social learning within organizations to curriculum delivery within a health service setting. Here, social participation is the basis of learning. We suggest that it offers a powerful framework for recognizing and explaining paradox and incongruence in clinical teaching and learning, and also for recognizing opportunities, and devising means, to add value to students’ learning experiences.”*

**References**

Lave, J., & Wenger, E. (1991). *Situated learning: Legitimate peripheral participation*. Cambridge: Cambridge University Press.

O'Brien, B. C., Battista, A. (2020). Situated learning theory in health professions education research: a scoping review. *Advances in Health Sciences Education, 25*, 483-509. doi: 10.1007/s10459-019-09900-w.

**Small Group Activity Handout with ‘Answer Key’**

**Applying Theoretical and Conceptual Frameworks – Case Vignette**

***Facilitator Note:*** *Participants are given the same case vignette and table, but their column titled ‘Situated Learning Theory’ is blank. Their goal is to read the description of SLT (above) followed by the Gap/Problem (below). Then, as a group they are to discuss the questions in each row to add answers in these spaces. The facilitator version below includes the ‘answer key’ to assist with this discussion. Some helpful guiding questions during the small group activity might be:*

- *Can someone describe SLT in their own words based on this description of it?*
- *What elements of narrative feedback [the problem] relate well to Situated Learning Theory based on the definition of SLT described here?*
- *Are there elements of narrative feedback that become less important when looking at it through the lens of SLT?*
- *What kinds of data might be helpful for ‘seeing’ SLT at work in narrative feedback?*

Gap/Problem: As part of a wide-ranging clinical curriculum reform, the University of Anywhere School of Medicine required preceptors to provide written narrative feedback to medical students. The school provided faculty with an app that allowed them to generate their comments on the fly. However, the curriculum administration soon noticed that the quality of feedback students received needed improvement. As a medical education researcher, Dr. Clark decided to conduct a study to investigate (and hopefully solve) this problem in an evidence-based way. From her literature review, she learned that good feedback is immediate, specific, corrective, and constructive. Also, the literature indicated that faculty development is critical to educate preceptors about feedback quality. However, it was not clear to Dr. Clark how faculty knowledge about feedback quality actually translates to improved written narrative feedback forms. She sought to identify the other factors that might be associated with producing quality written feedback in clinical teaching.

| Using the framework | Situated Learning Theory (SLT) |
| --- | --- |
| What research question will help you address the gap/problem in the opening role play (*see above summary*)? | How does the learning interaction between the preceptor and student impact narrative feedback quality (i.e., immediacy, specificity, corrective value, and constructiveness)? (*all groups will use the same research question*) |
| How would the research question be affected by using SLT as a lens for viewing the learning (and feedback) process? | Viewing learning through an SLT lens could affect the research question in multiple ways by prompting consideration of (among many possibilities):   - The role of feedback in facilitating deepened participation - The format of feedback needed to facilitate deepened participation - The social mechanisms operating on feedback delivery - The contextual factors that affect feedback delivery |
| In what areas of the project could SLT be used? Consider:   - Content (What to study?) - Methods (How?) - Interpretation (So what? What’s next?) | Content: Among many possibilities, SLT may prompt the researcher to study interactions between students and preceptors, identifying what kinds of interactions lead to better quality feedback. (Other content possibilities: SLT may prompt the researcher to investigate ways of defining feedback quality in terms of promoting deepening participation or to develop expanded ways of thinking about student-teacher-context roles in the feedback process.)  Methods: A study framed by SLT likely would use qualitative methods for observing (directly or indirectly) learner and preceptor interactions within a particular context (e.g., service teams on an internal medicine clerkship rotation).  Interpretation: Interpreting findings using SLT as a lens might focus on specifying the possibilities and limitations of written narrative feedback in allowing students to create, interpret, and reflect on learning experiences in the clinical context. |

Before concluding the small group, ask participants to share the following:

1. Questions they would like to bring to the large group
2. Tips/pearls they would want to share with others from what they learned today

*In addition, facilitators can remind participants that the list of references below (page 5) are provided in their handout and can be used for additional professional development on this topic.*

**Practical Approaches to Applying Conceptual & Theoretical Frameworks**

**to Medical Education Research**

Session References

1. Bordage G. Moving the field forward: going beyond quantitative-qualitative. Acad Med 2007; 82(10 SUPPL): S126-S128.
2. Eva KW, Lingard L. What’s next? A guiding question for educators engaged in educational research. Med Educ 2008; 42: 752-4.
3. Reeves S, Albert M, Kuper A, Hodges BD. Qualitative research: why use theories in qualitative research? BMJ 2008; 337: 631-4.
4. Bordage G. Conceptual frameworks to illuminate and magnify. Med Educ 2009; 43: 312-9.
5. Rocco TS, Plakhotnik MS. Literature reviews, conceptual frameworks, and theoretical frameworks: terms, functions, and distinctions. Hum Res Mgmt Rev 2009; 8: 120-9.
6. Bergman E, de Feijter J, Frambach J, Godefrooij M, Slootweg I, Stalmeijer R, van der Zwet J. AM last page: a guide to research paradigms relevant to medical education. Acad Med 2012; 87(4): 545.
7. Kusurkar R, ten Cate O. AM Last Page: Education is not filling a bucket, but lighting a fire: self-determination theory and motivation in medical students. Acad Med 2013; 88: 904.
8. Crites GE, Gaines JK, Cottrell S, Kalishman S, Gusic M, Mavis B, Durning SJ. Medical education scholarship: an introductory guide: AMEE Guide No. 89. Med Teach 2014; 36(8): 657-74.
9. Bierer SB, Foshee C, Uijtdehaage S. Strategies to remain current with the medical education field. Med Sci Educ 2015; 25(2): 163-70.
10. Bordage G, Lineberry M, Yudkowsky R. Conceptual frameworks to guide research and development (R&D) in health professions education. Acad Med 2016; 91(12): e2.
11. Dine CJ, Shea JA, Kogan JR. Generating good research questions in health professions education. Acad Med 2016; 91(12): e8.
12. Gottlieb M, Boysen-Osborn M, Chan TM, Krzyzaniak SM, Pineda N, Spector J, Sherbino J. Academic Primer Series: eight key papers about education theory. West J Emerg Med 2017; 18(2): 293-302.
13. Laksov B, Dornan T, Teunissen PW. Making theory explicit – an analysis of how medical education research(ers) describe how they connect to theory. BMC Med Ed 2017; 17: 18.
14. Lingard L. Writing an effective literature review. Perspect Med Educ 2018; 7: 47-9.
15. Meyer HS, Durning SJ, Sklar DP, Maggio LA. Making the first cut: an analysis of Academic Medicine editors’ reasons for not sending manuscripts out for external peer review. Acad Med 2018; 93(3): 464-70.
16. Zackoff MW, Real FJ, Abramson EL, Li ST, Klein MD, Gusic ME. Enhancing educational scholarship through conceptual frameworks: a challenge and roadmap for medical educators. Acad Pediatr 2019 Mar; 19(2): 135-41.
17. O'Brien BC, Battista A. Situated learning theory in health professions education research: a scoping review. Adv Health Sci Educ. 2020; 25: 483-509.
