# Supplementary material for: Applying Conceptual and Theoretical Frameworks to Health Professions Education Research: An Introductory Workshop
Source: MedEdPORTAL. 2022 Dec 2;18:11286. doi: 10.15766/mep_2374-8265.11286 (PMC9715823; doi:10.15766/mep_2374-8265.11286)
Supplement: Supplementary file 1 — Workshop Slides.pptxFacilitators’ Guide.docxParticipant Worksheet.docxWorkshop Evaluation.docx [file mep_2374-8265.11286-s001.zip › D. Workshop Evaluation.docx]

**WORKSHOP EVALUATION: Applying Conceptual & Theoretical Frameworks to Medical Education Research**

Please rate your ability to perform the following:

|  | Was able to do prior to the workshop | Am able to do as a result of the workshop | Unable to perform |
| --- | --- | --- | --- |
| Distinguish between conceptual frameworks, theories, and theoretical frameworks | o | o | o |
| Apply a conceptual or theoretical framework to a research question | o | o | o |
| Analyze how the selection of a conceptual or theoretical framework impacts research design | o | o | o |
| Discuss the results of a study through the lens of a conceptual or theoretical framework | o | o | o |

**For virtual workshop offerings only**

Please rate your level of engagement with the following:

|  | Highly engaged | Engaged | Neutral | Unengaged | Highly unengaged |
| --- | --- | --- | --- | --- | --- |
| Large group presentation | o | o | o | o | o |
| Breakout room discussion | o | o | o | o | o |

| What are the key messages / points you’ll take away? | How will you use them? |
| --- | --- |
| What did or did not work well, and why? | Please provide us with any additional comments about the session: |
