# Supplementary material for: Applying Conceptual and Theoretical Frameworks to Health Professions Education Research: An Introductory Workshop
Source: MedEdPORTAL. 2022 Dec 2;18:11286. doi: 10.15766/mep_2374-8265.11286 (PMC9715823; doi:10.15766/mep_2374-8265.11286)
Supplement: Supplementary file 1 — Workshop Slides.pptxFacilitators’ Guide.docxParticipant Worksheet.docxWorkshop Evaluation.docx [file mep_2374-8265.11286-s001.zip › Educational_Summary_Report_11286.pdf]

MedEdPORTAL® The AAMC Journal of Teaching and Learning Resources

Original Publication Open Access

Applying Conceptual and Theoretical Frameworks to Health Professions Education Research: An Introductory Workshop

Steven Rougas, MD, MS*, Andrea Berry, MPA, S. Beth Bierer, PhD, Rebecca D. Blanchard, PhD, Anna T. Cianciolo, PhD, Jorie M. Colbert-Getz, PhD, MS, Heeyoung Han, PhD, Kaitlin Lipner, MD, Cayla R. Teal, PhD

*Corresponding author: steven_rougas@brown.edu

Abstract

Introduction: Literature suggests that the quality and rigor of health professions education (HPE) research can be elevated if the research is anchored in existing theories and frameworks. This critical skill is difficult for novice researchers to master. We created a workshop to introduce the practical application of theories and frameworks to HPE research. **Methods:** We conducted two 60- to 75-minute workshops, one in 2019 at an in-person national conference and another in 2021 during an online national education conference. After a brief role-play introduction, participants applied a relevant theory to a case scenario in small groups, led by facilitators with expertise in HPE research. The workshop concluded with a presentation on applying the lessons learned when preparing a scholarly manuscript. We conducted a postworkshop survey to measure self-reported achievement of objectives. **Results:** Fifty-five individuals participated in the in-person workshop, and approximately 150 people completed the online workshop. Sixty participants (30%) completed the postworkshop survey across both workshops. As a result of participating in the workshop, 80% of participants (32) indicated they could distinguish between frameworks and theories, and 86% (32) could apply a conceptual or theoretical framework to a research question. Strengths of the workshop included the small-group activity, access to expert facilitators, and the materials provided. **Discussion:** The workshop has been well received by participants and fills a gap in the existing resources available to HPE researchers and mentors. It can be replicated in multiple settings to model the application of conceptual and theoretical frameworks to HPE research.

Keywords
Conceptual Framework, Theoretical Framework, Theory, Continuing Professional Development, Faculty Development, Publishing/Scholarship

Educational Objectives

By the end of this activity, learners will be able to:

1. Describe conceptual and theoretical frameworks commonly used in health professions education research.
2. Examine how the selection of a framework affects research design.
3. Discuss strategies for presenting results relative to a conceptual or theoretical framework.

Citation:
Rougas S, Berry A, Bierer SB, et al. Applying conceptual and theoretical frameworks to health professions education research: an introductory workshop. *MedEdPORTAL*. 2022;18:11286.
https://doi.org/10.15766/mep_2374-8265.11286

Introduction

Calls for improved rigor in health professions education (HPE) research have often focused on the need to incorporate theoretical and conceptual frameworks in research design, implementation, and reflective critique.[1,2] Theories, which explain how/why things are related to each other, and frameworks, which explain where a study originates and the implications on study design, are critical for conducting high-quality HPE research, yet many researchers struggle to apply them.[3] Ideally, conceptual or theoretical frameworks should provide a lens through which to identify gaps in the literature, operationalize constructs, hypothesize relationships, and design appropriate methodology.[4] Frameworks allow researchers to deepen their understanding of how societies, organizations, and people interact[5] and can help HPE researchers engage in the adequate preparation needed for a scholarly inquiry.[6]

A robust literature emphasizes the importance of anchoring HPE research in existing theories and frameworks.[7-9] Frameworks

Copyright © 2022 Rougas et al. This is an open-access publication distributed under the terms of the Creative Commons Attribution license.

ideally should be used early to influence the what (content) and the how (methodology) of a research project and then revisited to help situate the results.[10] Recent attention to terminology[11] and application[1,12,13] has provided additional resources to support HPE researchers. Yet selection and application of a suitable conceptual or theoretical framework are still underutilized, and the lack of such frameworks is a common reason for manuscript rejection in major HPE journals.[14]

One reason for poor utilization may be a lack of consensus on how HPE researchers define theory, theoretical framework, and conceptual framework.[11] Despite references to conceptual and theoretical frameworks in reviews of manuscripts and grant submissions, there is a surprising absence of consistency in how these terms are used. After a review of relevant literature, we agreed upon the following focused definitions to guide our work:

1. Theory: an explanation of how/why things are related to each other.
2. Theoretical framework: the implications of the theory for study design.
3. Conceptual framework: the conceptual heritage (i.e., the central concepts used in a field of study) of the problem to be studied.

Another reason for poor utilization is inconsistent application of these concepts. The volume of theoretical and conceptual frameworks applicable to HPE research can be overwhelming,[15] and researchers often see framework selection as the end product of their effort rather than an initial step. The framework should resonate with the researcher and the conceptual heritage of the project[16] and be used in every part of the research process from development of the research question and methodology to analysis of the results and discussion of study findings.[12,13] Researchers often lose sight of this guiding principle once the theory or framework is selected.

A final reason may be the fact that many educators have received minimal training in HPE research, particularly the incorporation of conceptual or theoretical frameworks to guide such work. While faculty development programs have begun to address this need, the majority of such programs still tend to focus on teaching and learning topics.[17] To improve HPE research quality, considerable training in research methods must occur.[18] Though various workshops exist to expose HPE researchers to principles of scholarly writing,[19] method design,[20] statistics,[21] and academic career development,[22] there remains a gap in the knowledge and skills needed to apply conceptual and theoretical frameworks.

As members of the AAMC's Medical Education Scholarship Research and Evaluation (MESRE) section of the Group on Educational Affairs (GEA) who provide mentorship, consultation, and critical review for various HPE research projects locally, regionally, and nationally, we recognized the need for a structured professional development opportunity for novice researchers to learn application of these concepts. The goal of our project was to develop an interactive, case-based workshop to explore the application of conceptual and theoretical frameworks to HPE research.

Methods

Workshop Design

We designed this workshop for HPE researchers seeking guidance on how to apply conceptual and theoretical frameworks. The workshop included an introductory video role-play, small-group discussion of a case, and large-group debriefing. Although the case scenario used in the workshop featured educators studying written narrative feedback in undergraduate medical education, the workshop could appeal to any HPE researcher wishing to gain experience with using conceptual and theoretical frameworks. Participants did not need prerequisite knowledge of theories in order to achieve the workshop objectives.

The workshop planners included educators serving on the MESRE steering committee in 2019. All had experience mentoring others on how to use conceptual and theoretical frameworks, and a subset served on HPE journal editorial boards or as peer reviewers. We designed workshop materials and participated in the workshop as a featured speaker and/or small-group facilitator.

We developed the workshop to be offered in person at a conference and later adapted it to be presented virtually. We initially designed the workshop as a 75-minute session but learned, during the second offering, that 60 minutes provided sufficient time to meet the workshop objectives. When offering the workshop in a face-to-face venue, we used a large conference room with projection equipment and internet access to display slides and videos. The conference room had to have enough tables for participants to work in small groups of five to 10. When conducting the session virtually, we selected a platform that enabled the workshop facilitator to assign participants to breakout rooms and permitted small-group facilitators to share their screens with workshop participants.

We had a main facilitator for the workshop and several small-group facilitators for the small-group work. All facilitators

Copyright © 2022 Rougas et al. This is an open-access publication distributed under the terms of the Creative Commons Attribution license.

reviewed information on situated learning theory (SLT), as this theory was used in the workshop case scenario. Facilitators also needed experience with using theory to inform the elaboration of research questions, the design of research projects, and the interpretation of research findings. Ideally, facilitators had experience with publishing peer-reviewed manuscripts including conceptual or theoretical frameworks and came from any HPE field. While it would be possible to run the workshop without all these essential skills, we highly recommend recruiting small-group facilitators with them. We also advise having at least one facilitator for every 10 workshop participants. The lesson plan and timeline for the workshop are outlined in Table 1.

Preworkshop Preparation

We conducted a training session (approximately 45-60 minutes) with small-group facilitators prior to the workshop to review the workshop slides (Appendix A) and facilitation strategies for the case discussion (Appendix B).

Introduction (2 Minutes)

When feasible, participants were asked to introduce themselves at the beginning of the workshop. Following introductions, the workshop's learning objectives were presented. We kept the workshop overview short, as the first video vignette contextualized the workshop topic.

Video Vignette 1 (5 Minutes)

After introducing the session objectives, the main facilitator played a recorded role-play (Appendix A, slide 4) demonstrating a conversation between a mentor and mentee about the importance of selecting a theory to guide the design of a research project. This case scenario provided participants with an authentic example emphasizing the importance of selecting an appropriate theory to publish a research project in HPE journals.

Define Key Terms (2 Minutes)

The main facilitator proceeded to define the key terms—conceptual framework, theory, and theoretical framework—mentioned in the role-play (Appendix A, slides 5-6). Participants may not have fully recognized the distinction between a theoretical framework and conceptual framework at this point in the workshop.

Video Vignette 2 (3 Minutes)

The main facilitator showed a second recording of the role-play (Appendix A, slide 7) to illustrate how theory contributes to the interpretation of key findings and, after the video, discussed how one's point of view could frame the ways in which one examined problems (Appendix A, slides 8-11). Building upon the earlier case vignette scenario, the main facilitator presented a worked example to demonstrate how the theory of planned behavior could apply to the feedback study featured in the video vignette (Appendix A, slides 12-13).

Small-Group Activity (25-30 Minutes)

Participants formed small groups of five to 10 (or joined breakout rooms) for a 30-minute small-group activity designed to apply a different theory to the case scenario. Small-group facilitators began the session by reviewing the key features of SLT summarized on the participant worksheet (Appendix C). Subsequently, small-group facilitators guided the small groups to think about how the research question provided could be refined using SLT and helped them consider how SLT could guide research design including study participants, setting, data sources, and data collection strategies. Before the small-group activity concluded, each small-group facilitator asked about any remaining questions or tips the participants wanted to share with the larger group and recorded these responses for subsequent large-group discussion.

Debriefing and Wrap-up (12 Minutes)

The main facilitator reconvened all small groups and reviewed guiding questions participants had discussed during the small-group activity. Small-group facilitators presented the remaining questions and tips their groups had identified. The main facilitator then concluded by presenting a journal editor's discussion of

Table 1. Recommended Timeline for the Session

Activity	Resource	Allotted Time
Introductions and workshop objectives	Appendix A: slides 1-3	2 minutes
Present video vignette 1 of role-play demonstrating how to coach a person about the importance of conceptual and theoretical frameworks	Appendix A: slide 4	5 minutes
Define key terms	Appendix A: slides 5-6	2 minutes
Present video vignette 2 of role-play demonstrating importance of using theory to interpret research findings	Appendix A: slide 7	3 minutes
Discuss how various points of view frame how to examine problems; present a worked example using the theory of planned behavior	Appendix A: slides 8-13	5 minutes
Small-group activity	Appendices B and C	25-30 minutes
Debrief small-group activity		5 minutes
Discuss application of theory to frame research projects	Appendix A: slides 15-19	5 minutes
Workshop evaluation and wrap-up	Appendix D	2 minutes

Copyright © 2022 Rougas et al. This is an open-access publication distributed under the terms of the Creative Commons Attribution license.

how these concepts applied to manuscripts and editorial review (Appendix A, slides 15-19). Participants completed a workshop evaluation (Appendix D) at the end of the session.

Workshop Evaluation and Analysis

We developed the workshop evaluation (Appendix D) to assess the workshop's effectiveness and gather information to improve the workshop. For effectiveness questions, participants used a 3-point scale ("was able to do prior to workshop," "am able to do as a result of the workshop," and "unable to perform") to rate their ability to perform workshop objectives. The workshop evaluation included four open-ended items ("What are the key points/messages you will take away?", "How will you use them?", "What did or did not work well and why?", and "Please provide us with any additional comments about the session") to identify strengths of the workshop as well as areas for improvement. For the virtual session, we also asked participants to rate their level of engagement with the large-group and breakout-room components. We calculated frequencies for scaled items using Microsoft Excel and analyzed written comments for major themes. We were particularly interested in determining the percentage of participants who could meet each workshop objective as a result of the workshop versus those who could not meet the objective; therefore, we included only those who selected these options in the denominator of the frequency calculation. Those who could meet an objective prior to the workshop were excluded from the calculations, as our goal was to guide those who lacked a skill prior to attending the workshop. We used a paper evaluation form for the face-to-face offering and an electronic form for the virtual session. We also included an item on engagement for the virtual offering, as there were fewer cues provided to facilitators in the online setting.

Results

The workshop was delivered in person at Learn Serve Lead: the AAMC Annual Meeting in November 2019 and virtually at the GEA Regional Spring Meeting in April 2021. Workshop participants were medical school staff, faculty, and administrators. Fifty-five attendees participated in the in-person workshop, and approximately 150 participants participated in the virtual workshop. The postworkshop survey was completed by 26 individuals from the in-person session (47%) and 34 from the virtual session (23%).

Table 2 provides the frequency of participants' self-reported ability to perform the workshop objectives relative to not being able to perform them. As a result of participating in the workshop, 80% of the included participants (32) indicated they could distinguish between conceptual frameworks and theories, 86% (32) could apply a conceptual or theoretical framework to a research question, 79% (34) could analyze how the selection of a conceptual or theoretical framework impacts research design, and 68% (27) could evaluate the results of a study through the lens of a conceptual or theoretical framework (Figure).

Participant comments on what did and did not work well and why were categorized by workshop elements, and frequencies for each element were computed based on terms referring to strengths or areas for improvement. The majority of comments highlighted strengths of the workshop, including the small-group/breakout activity (12 participants), having expert facilitators (nine participants), the role-play (seven participants), and the handouts (seven participants). Other positive aspects were the large-group discussion (four participants), use of examples (four participants), and time for questions (two participants). Participants felt the small-group activity, expert facilitators, and role-playing worked well because they were active, hands-on activities. A few participants mentioned areas for improvement, including small-group variability (seven participants), additional terms needing definitions (four participants), overall complexity of the topic (two participants), and wanting more resources (two participants).

Thirty-four virtual workshop participants rated the overall engagement of the large-group presentation and small-group (breakout-room) discussion. The large-group presentation was rated as highly engaging (53%, 18) or engaging (35%, 12) by 88%

Table 2. Participants' Frequency of Being Able to Perform Each Objective Prior to the Workshop or as a Result of the Workshop or Being Unable to Perform

Workshop Objective	Was Able to Do Prior to the Workshop	Am Able to Do as a Result of the Workshop	Unable to Perform
Distinguish between conceptual frameworks and theories (n = 59)	19	32	8
Apply a conceptual or theoretical framework to a research question (n = 60)	23	32	5
Analyze how the selection of a conceptual or theoretical framework impacts research design (n = 58)	15	34	9
Evaluate the results of a study through the lens of a conceptual or theoretical framework (n = 59)	19	27	13

Copyright © 2022 Rougas et al. This is an open-access publication distributed under the terms of the Creative Commons Attribution license.

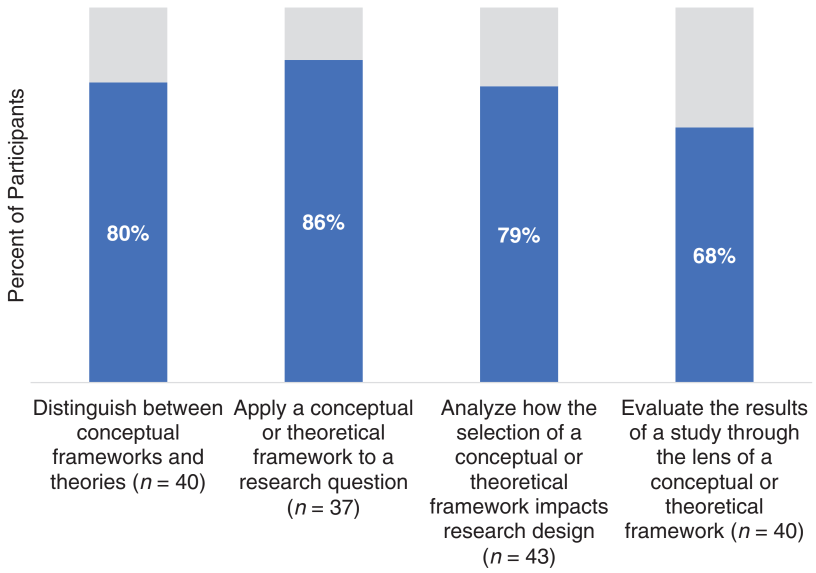

Figure. Percentage of participants reporting the ability to perform each workshop objective as a result of the workshop relative to not being able to perform the objective.

of participants. The small-group discussion was rated as highly engaging (71%, 24) or engaging (9%, 3) by 80% of participants.

Discussion

This workshop enabled participants to work in small groups with a facilitator to apply a theory to an example research question and discuss the implications of the effort. The workshop was well received by participants, who mostly reported self-improvement regarding each of the learning objectives and valued the small-group activity, work with facilitators, and observation of the role-play. For facilitators, the workshop offered a unique opportunity to focus teaching on the application of a theoretical or conceptual framework, separate from other aspects of HPE research mentorship. The materials included here provide an accessible avenue for HPE research champions to engage novice researchers at their own institutions in a skill that is challenging to learn and to teach.

Our reflections presented valuable insight on the preparation and execution of a workshop designed to teach about a complex and typically unfamiliar topic. While careful selection of a facilitator and thoughtful preparation of materials generally result in better experiences for learners, in this workshop, those details are particularly important.

The workshop has been designed for novice HPE researchers. As a result, learner questions can be oversimplified, while the answers are nuanced. Facilitators must be able to navigate these conversations as well as hold reasonable goals for the development of learners on this complex topic. Appropriate facilitators for this workshop include those who are comfortable using frameworks in their own research and coaching/mentoring others. Being familiar with more than one theory or conceptual framework that could apply to the case scenario is particularly valuable, as the ability to compare and contrast frameworks is helpful for answering questions and reframing complex answers in an accessible way. Conducting the workshop virtually may assist in recruiting qualified small-group facilitators from other departments or institutions. Prior to each session, facilitators should meet virtually to discuss the facilitators' guide (Appendix B). This allows for discussion of the case vignette and consensus on how to approach potential questions or roadblocks.

We found that small groups struggling to address the discussion questions often did not spend an adequate amount of time exploring SLT in the beginning of the small-group activity. Though we provided a paragraph of text and references describing the theory (Appendices B and C), a visual schema could simplify some of the connections and give novice researchers a clearer, more succinct way into the conversation. One potential approach is to use some of the small-group time to develop the schema with participants.

Similarly, comparing the application of one theory to another provides insight into how a study can be viewed through multiple lenses. While many of our facilitators employed this approach, it proved challenging given the timing. One potential approach is to

Copyright © 2022 Rougas et al. This is an open-access publication distributed under the terms of the Creative Commons Attribution license.

use the theory of planned behavior (reviewed in the introductory slides, Appendix A) as the comparative theory since it has already been referenced.

The small-group exercise was rated the most valuable. Discussions and pacing varied due to participants' knowledge and comfort with speaking up and facilitators' ability to manage the conversation. Therefore, we recommend ensuring adequate time for completing the small-group activity. Specifically, we recommend facilitators be mindful of how much time is allotted to reviewing the theory compared to the amount allotted for the discussion. We suggest sending out the background information on SLT in advance, so that participants have a chance to review the key information prior to the workshop.

We also suggest tailoring the description of the workshop and learning objectives in order to invite the appropriate audience. This may mean novice researchers who have engaged with research or who are currently planning research. Participants do not need to have the same level of knowledge entering the workshop, but because of the emphasis on small-group discussion, wide variation in participants' knowledge may result in some not obtaining the insight they need to meet the learning objectives.

A common refrain from workshop participants was how to select the right answers to the discussion questions rather than trying to understand how the theory selected impacted the research question. This likely stemmed from a common misconception that there was only one right theory or framework for a research study. Confronting this expectation early in the session (in both the introduction and the small-group activity) is key.

Workshops are inherently limiting in that they can accommodate only a small number of learners. However, given the complexity of this topic, the small number of learners may improve the experience, as conversation can be guided towards specific learner gaps.

This workshop has additional limitations; however, with thoughtful preparation, they can be addressed to ensure a valuable learning experience. First, the workshop requires strong facilitators, which limits workshop size for institutions without access to experienced facilitators. Second, the amount of time needed to complete the workshop requires facilitators to pay careful attention to workshop timing. Third, small-group experiences can vary considerably with facilitator expertise and familiarity with SLT. Thoughtful recruitment and training of facilitators, perhaps relying on experienced facilitators to train new ones, will maximize participant benefit.

Finally, the evaluation data we collected postsession did not adequately account for knowledge or skills that participants had prior to the workshop. While the anecdotal feedback was that most participants were not very knowledgeable or skilled, a pre/post design would have helped clarify this issue. The rating scale forced participants to mark effectiveness items as being performable either prior to or as a result of the workshop. This did not take into account that some participants had prior knowledge or skill and still benefited from the workshop, making interpretation of the responses less clear. Additionally, the evaluation response rate was low, especially for the virtual session, and may not represent the perspectives of all participants.

This workshop has the potential to increase the application of theories and frameworks in HPE research. Frameworks are helpful to organize studies in the context of a greater conversation but are difficult to learn outside of formal educational programs. The workshop enables novice HPE researchers to explore how they might begin integrating frameworks into their work and why doing so is important.

The workshop provides scaffolding for HPE research mentors to introduce frameworks to novice and emerging researchers, and the materials included constitute a valuable reference. In addition, workshops like this one provide support and structure for institutions with few HPE research mentors. Future directions should focus on increasing accessibility of this information to more HPE researchers through the creation of an interactive, online session and a searchable repository of theories and frameworks commonly used in HPE research.

Appendices

A. Workshop Slides.pptx

B. Facilitators' Guide.docx

C. Participant Worksheet.docx

D. Workshop Evaluation.docx

All appendices are peer reviewed as integral parts of the Original Publication.

Steven Rougas, MD, MS: Associate Professor of Emergency Medicine and Medical Science and Director, Doctoring Program, Warren Alpert Medical School of Brown University; ORCID: https://orcid.org/0000-0003-2225-9657

Copyright © 2022 Rougas et al. This is an open-access publication distributed under the terms of the Creative Commons Attribution license.

Andrea Berry, MPA: Executive Director of Faculty Life, University of Central Florida College of Medicine

S. Beth Bierer, PhD: Director of Assessment and Evaluation and Professor of Medicine, Cleveland Clinic Lerner College of Medicine of Case Western Reserve University

Rebecca D. Blanchard, PhD: Director of Faculty Development, OnlineMedEd, and Associate Professor, Department of Medicine, University of Massachusetts Medical School-Baystate

Anna T. Cianciolo, PhD: Associate Professor, Department of Medical Education, Southern Illinois University School of Medicine

Jorie M. Colbert-Getz, PhD, MS: Assistant Dean of Education Quality Improvement and Associate Professor, Department of Internal Medicine, University of Utah School of Medicine

Heeyoung Han, PhD: Associate Professor and Director of Postdoctoral Program, Department of Medical Education, Southern Illinois University School of Medicine

Kaitlin Lipner, MD: Second-Year Resident, Department of Emergency Medicine, Warren Alpert Medical School of Brown University

Cayla R. Teal, PhD: Associate Dean for Assessment and Evaluation and Education Associate Professor of Population Health, University of Kansas School of Medicine

Disclosures
None to report.

Funding/Support
None to report.

Prior Presentations
Rougas S, Berry A, Bierer B, et al. Practical approaches to applying conceptual and theoretical frameworks to medical education research: a MESRE session. Presented at: Learn Serve Lead: the AAMC Annual Meeting; November 8-12, 2019; Phoenix, AZ.

Rougas S, Berry A, Bierer B, et al. Practical approaches to applying conceptual and theoretical frameworks to medical education research. Presented virtually at: Group on Educational Affairs Regional Spring Meeting; April 20-22, 2021.

Ethical Approval
Reported as not applicable.

References

1. Zackoff MW, Real FJ, Abramson EL, Li STT, Klein MD, Gusic ME. Enhancing educational scholarship through conceptual frameworks: a challenge and roadmap for medical educators. *Acad Pediatr*. 2019;19(2):135-141. https://doi.org/10.1016/j.acap.2018.08.003
2. Crites GE, Gaines JK, Cottrell S, et al. Medical education scholarship: an introductory guide: AMEE Guide no. 89. *Med Teach*. 2014;36(8):657-674. https://doi.org/10.3109/0142159X.2014.916791
3. O'Sullivan P, Uijtdehaage S, Kalishman S, et al. Example conceptual frameworks: to guide educational scholarship. Presented at: Teaching Academy of the Consortium of Western Regional Colleges of Veterinary Medicine; July 2013; Corvallis, OR.
4. Bordage G. Conceptual frameworks to illuminate and magnify. *Med Educ*. 2009;43(4):312-319. https://doi.org/10.1111/j.1365-2923.2009.03295.x
5. Reeves S, Albert M, Kuper A, Hodges BD. Why use theories in qualitative research? *BMJ*. 2008;337:a949. https://doi.org/10.1136/bmj.a949
6. Glassick CE, Huber MT, Maeroff GI. *Scholarship Assessed: Evaluation of the Professoriate*. Jossey-Bass; 1997.
7. Sandars J, Cleary TJ. Self-regulation theory: applications to medical education: AMEE Guide no. 58. *Med Teach*. 2011;33(11):875-886. https://doi.org/10.3109/0142159X.2011.595434
8. Schuwirth LWT, Van der Vleuten CPM. Programmatic assessment: from assessment of learning to assessment for learning. *Med Teach*. 2011;33(6):478-485. https://doi.org/10.3109/0142159X.2011.565828
9. Yardley S, Teunissen PW, Dornan T. Experiential learning: transforming theory into practice. *Med Teach*. 2012;34(2):161-164. https://doi.org/10.3109/0142159X.2012.643264
10. Bordage G. Moving the field forward: going beyond quantitative–qualitative. *Acad Med*. 2007;82(suppl 10):S126-S128. https://doi.org/10.1097/ACM.0b013e31813e661d
11. Varpio L, Paradis E, Uijtdehaage S, Young M. The distinctions between theory, theoretical framework, and conceptual framework. *Acad Med*. 2019;95(7):989-994. https://doi.org/10.1097/ACM.0000000000003075
12. Laksov KB, Dornan T, Teunissen PW. Making theory explicit—an analysis of how medical education research(ers) describe how they connect to theory. *BMC Med Educ*. 2017;17:18. https://doi.org/10.1186/s12909-016-0848-1
13. Samuel A, Konopasky A, Schuwirth LWT, King SM, Durning SJ. Five principles for using educational theory: strategies for advancing health professions education research. *Acad Med*. 2020;95(4):518-522. https://doi.org/10.1097/ACM.0000000000003066
14. Meyer HS, Durning SJ, Sklar DP, Maggio LA. Making the first cut: an analysis of *Academic Medicine* editors' reasons for not sending manuscripts out for external peer review. *Acad Med*. 2018;93(3):464-470. https://doi.org/10.1097/ACM.0000000000001860
15. Millwood R. Learning theory. Holistic Approach to Technology Enhanced Learning. April 30, 2013. Accessed October 11, 2022.

Copyright © 2022 Rougas et al. This is an open-access publication distributed under the terms of the Creative Commons Attribution license.

http://hotel-project.eu/sites/default/files/hotel/default/content-files/documentation/Learning-Theory.pdf

16. Mann KV. Theoretical perspectives in medical education: past experience and future possibilities. *Med Educ*. 2011;45(1):60-68. https://doi.org/10.1111/j.1365-2923.2010.03757.x
17. LaMantia J, Hamstra SJ, Martin DR, et al. Faculty development in medical education research. *Acad Emerg Med*. 2012;19(12):1462-1467. https://doi.org/10.1111/acem.12037
18. Gruppen LD. Improving medical education research. *Teach Learn Med*. 2007;19(4):331-335. https://doi.org/10.1080/10401330701542370
19. Li STT, Gusic ME, Vinci RJ, Szilagyi PG, Klein MD. A structured framework and resources to use to get your medical education work published. *MedEdPORTAL*. 2018;14:10669. https://doi.org/10.15766/mep_2374-8265.10669
20. Ryan MS, Quigley PD, Lee CC, et al. Innovation to dissemination workshop: selecting outcome measures to translate educational innovations into scholarship. *MedEdPORTAL*. 2018;14:10759. https://doi.org/10.15766/mep_2374-8265.10759
21. Windish DM. A guide to basic statistics for educational research. *MedEdPORTAL*. 2021;17:11187. https://doi.org/10.15766/mep_2374-8265.11187
22. Paredes Molina CS, Spencer DJ, Morcuende M, et al. An introduction to research work, scholarship, and paving a way to a career in academic medicine. *MedEdPORTAL*. 2018;14:10686. https://doi.org/10.15766/mep_2374-8265.10686

Received: April 7, 2022
Accepted: August 31, 2022
Published: December 2, 2022

Copyright © 2022 Rougas et al. This is an open-access publication distributed under the terms of the Creative Commons Attribution license.
